# Large contribution of in-cloud production of secondary organic aerosol from biomass burning emissions

**DOI:** 10.1038/s41612-024-00682-6

**Published:** 2024-06-25

**Authors:** Tiantian Wang, Kun Li, David M. Bell, Jun Zhang, Tianqu Cui, Mihnea Surdu, Urs Baltensperger, Jay G. Slowik, Houssni Lamkaddam, Imad El Haddad, Andre S. H. Prevot

**Affiliations:** 1 PSI Center for Energy and Environmental Sciences, Paul Scherrer Institute, 5232 Villigen, Switzerland; 2 Present Address: Environmental Research Institute, Shandong University, Qingdao, 266237 China

**Keywords:** Atmospheric chemistry, Environmental impact

## Abstract

Organic compounds released from wildfires and residential biomass burning play a crucial role in shaping the composition of the atmosphere. The solubility and subsequent reactions of these compounds in the aqueous phase of clouds and fog remain poorly understood. Nevertheless, these compounds have the potential to become an important source of secondary organic aerosol (SOA). In this study, we simulated the aqueous SOA (aqSOA) from residential wood burning emissions under atmospherically relevant conditions of gas-liquid phase partitioning, using a wetted-wall flow reactor (WFR). We analyzed and quantified the specific compounds present in these emissions at a molecular level and determined their solubility in clouds. Our findings reveal that while 1% of organic compounds are fully water-soluble, 19% exhibit moderate solubility and can partition into the aqueous phase in a thick cloud. Furthermore, it is found that the aqSOA generated in our laboratory experiments has a substantial fraction being attributed to the formation of oligomers in the aqueous phase. We also determined an aqSOA yield of 20% from residential wood combustion, which surpasses current estimates based on gas-phase oxidation. These results indicate that in-cloud chemistry of organic gases emitted from wood burning can serve as an efficient pathway to produce organic aerosols, thus potentially influencing climate and air quality.

## Introduction

Organic aerosol (OA) accounts for a substantial mass fraction of submicron aerosol^1^. Understanding OA sources is a critical environmental challenge that is required to reduce some of the larger uncertainties in assessing radiative climate forcing and air quality management policies^2–4^. OA consists of primary organic aerosol (POA), directly emitted into the atmosphere, and secondary organic aerosol (SOA), formed via the conversion of volatile organic compounds (VOCs) into low-volatility species. Bottom-up and top-down estimates confirm that SOA accounts for up to 76% of ambient OA^5–7^. Over the past decades, our knowledge of SOA production via gas-phase chemistry has greatly improved, whereas SOA production via in-cloud chemistry has received far less attention. Water-soluble organic gases (WSOGs) can react in aqueous media to form highly oxygenated, low-volatility, aqueous SOA (aqSOA)^8–11^. Notably, during haze episodes in China and foggy periods in the Po Valley, remarkably high concentrations of aqSOA have been observed^10,12^. Although it is expected that biomass burning emissions are present during these events, the vapors contributing to aqueous SOA production during these events remain unknown. Biomass burning (BB) emissions, including those from residential burning, represent the second largest global source of non-methane organic gases (NMOGs) after biogenic emissions^13,14^. Despite advancements in the chemical characterization of organic gases and a comprehensive summary of Henry’s law constants^15–17^, the water solubility and potential contribution of BB gases to aqSOA formation remain unknown. BB emissions consist of numerous compounds, many of which could play a role in aqSOA formation^18^. Consequently, the impact of complex primary BB emissions on SOA formation remains largely understudied, necessitating a holistic characterization and quantification.

Because of technological limitations, typical laboratory experiments fail to mimic aqSOA formation from in-cloud processes under atmospherically relevant conditions. Cloud chambers in laboratories produce clouds with extremely short lifetimes (a few minutes), while bulk solution experiments overestimate the liquid water content (LWC) in the atmosphere by at least six orders of magnitude (10^6^ g m^−3^ in bulk water vs. 1 g m^−3^ in thick clouds). Furthermore, the current literature on aqSOA formation primarily focuses on individual dissolved compounds (e.g., glycolaldehyde, acetic acid, levoglucosan, phenol, vanillin, and guaiacol)^19–22^. However, there is a lack of studies on aqSOA formation from complex mixtures of WSOGs from real-world biomass burning emissions. The highly variable nature of biomass burning emissions poses a great challenge for their investigation using continuous flow reactors.

To overcome these limitations, a wetted-wall flow reactor (WFR) was used in combination with a holding tank to simulate in-cloud production of aqSOA from residential wood burning emissions. The WFR, previously utilized for investigating isoprene cloud chemistry with an atmospherically relevant aqueous solution composition^23^, was applied to a system of complex emissions. The WFR was maintained at approximately 100% relative humidity (RH), while organic gases from residential wood burning were introduced into the WFR along with OH radicals and water vapor under kinetic conditions. The gas-phase chemical composition was quantified and characterized using a suite of mass spectrometers. We determined the water solubility of biomass burning vapors based on their uptake into the water layer. Compounds formed through aqueous-phase oxidation in the WFR were nebulized to determine the yields, bulk chemical composition and molecular composition of the resulting aqSOA. We compared our laboratory findings with the chemical composition of gasSOA from the same BB emissions.

## Results

### Molecular composition of gaseous organic compounds from primary residential wood burning emissions

**Fig. 1:**
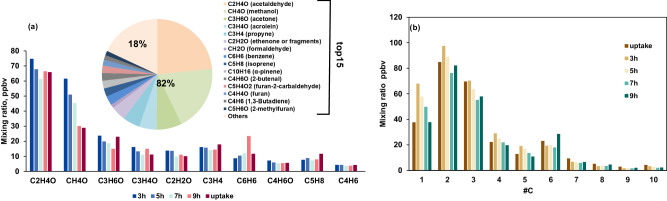
Main VOCs from biomass burning measured by PTR-MS. **a** Concentrations of the 10 most abundant VOCs (or VOC fragments) measured by the PTR-MS in wood burning primary emissions, where the bar colors denote the different experiments in the WFR. The pie chart presents the average contribution of mixing ratios in one wood burning experiment. **b** Carbon number distributions of wood burning primary emissions measured by the PTR-MS for the different WFR experiments.

Previous online measurements of organic gases from residential wood burning primarily utilized a proton transfer reaction mass spectrometer (PTR-MS), and over 85% of the measured NMOG mass from residential wood combustion emissions have been identified, which provides assigned molecular formulae and structural information to observed ions using the PTR-MS^24,25^. As shown in Fig. 1, the 15 species with the highest concentration as measured by the PTR-MS are structurally assigned based on previously identified residential wood combustion products, which account for roughly 80% of the total NMOGs across our entire experiment sets and belong to the VOC volatility class (log_10_ (C*) > 6.5 μg m^−3^). However, in previous studies intermediate-volatility organic compounds (IVOCs, 2.5 μg m^−3^< log_10_ (C*) < 6.5 μg m^−3^) were likely not detected by the PTR-MS. To measure the IVOCs, a Vocus PTR-TOF-MS (Tofwerk; hereafter referred to as Vocus) and a dual-phase extractive electrospray ionization time-of-flight mass spectrometer (Dual-EESI for short) (Tofwerk/Aerodyne) were also used^26^. The combination of the Vocus and Dual-EESI detected 680 elemental formulae between *m/z* 40 and *m/z* 350 (Supplementary Fig. 1). The IVOCs identified by both instruments contributed approximately 15.7% of the total vapor concentration.

**Fig. 2:**
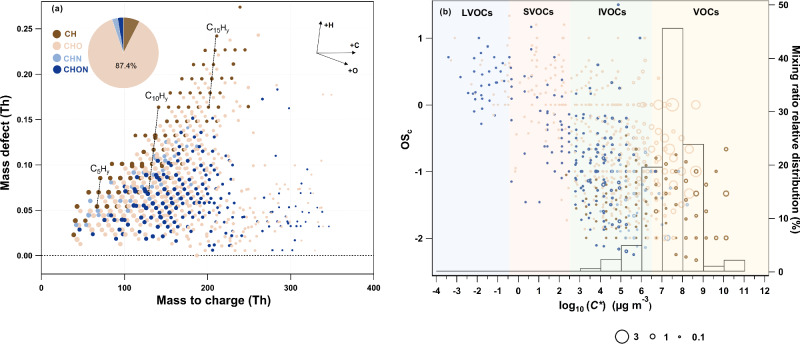
Measured gas-phase composition from residential wood burning. **a** Mass-defect plot of the ions identified by high-resolution analysis of the Vocus and Dual-EESI data sets. Data points are color-coded by ion family (CH, CHO, CHN, CHON) and sized by the logarithm of their concentration. **b** Two-dimensional volatility basis set (2D-VBS) of primary biomass burning products and volatility distribution. Compounds are plotted as carbon oxidation states (OS_C_, calculated as 2 × *O/C* – *H/C*) versus estimated volatility (log_10_ (C*)). Markers are colored according to ion family and sized by the square root of their fractional contribution. Shaded areas indicate the volatility ranges of low-volatility (LVOC, log_10_ (C*) < −0.5 μg m^−3^, orange), semi-volatile (SVOC, −0.5 μg m^−3^ < log_10_ (C*) < 2.5 μg m^−3^, green), intermediate-volatility (IVOC, 2.5 μg m^−3^< log_10_ (C*) < 6.5 μg m^−3^, blue) and volatile organic compounds (VOC, log_10_ (C*) > 6.5 μg m^−3^, yellow).

The gas-phase analysis revealed several dominant species: C_2_H_4_O_2_ (acetic acid, 10.4% of the total concentration), C_2_H_4_O (acetaldehyde, 7%), C_3_H_6_O_2_ (methyl acetate, 6.1%), C_3_H_6_O (acetone, 5.8%), and C_5_H_4_O_2_ (furfural, 5.5%). Figure 2a presents the mass defect plot of organic compounds grouped into different ion families from a representative experiment, including CH, CHO, CHN and CHON compounds. The volatilities, represented by the saturation vapor concentrations (log_10_ (C*) μg m^−3^), of all organic compounds were predicted as a function of elemental composition as determined by the Vocus and Dual-EESI, using the parameterization by Li et al.^27^. Figure 2b displays the volatility distribution as a function of the carbon oxidation state (OSc, calculated as 2 × *O/C* – *H/C*). Low-volatility compounds are predominantly in the CHO and CHON families, while high-volatility compounds fall into the CH family. The CHO group accounts for the largest fraction of the total primary organic gases (87.4% of the total mixing ratio), followed by the CH (7.5%), CHN (2.6%) and CHON (2.5%). CHO compounds are present across a large volatility range, with 14% assigned to the IVOCs and semi-volatile organic compounds (SVOCs, −0.5 μg m^−3^ < log_10_ (C*) < 2.5 μg m^−3^).

### Water solubility of biomass burning organic vapors

After the characterization of the primary gas-phase BB emissions, Xe-excimer lamps were switched on to generate OH radicals (Supplementary Fig. 2)^28^. A slight decrease ( ~ 9.8%) in the concentrations of the total primary organic gases was observed due to their consumption by OH radicals, and a corresponding increase of oxygenated compounds was observed (Supplementary Fig. 5c). Once a relatively steady state was reached, we simulated the presence of a cloud experimentally by introducing liquid water into the WFR, resulting in the formation of an aqueous microlayer on the reactor wall. A considerable decrease of the concentrations of some gas-phase species occurred, due to their uptake into the aqueous microlayer. Representative time series of species with different solubility are depicted in Supplementary Fig. 6. The partitioning of gas-phase species into the aqueous phase depends on their bulk solubility. Non-soluble compounds (e.g., C_6_H_8_, 1,3-cyclohexadiene or fragments) remain unperturbed, while soluble compounds (e.g., C_4_H_6_O_4_) experience a continuous and irreversible uptake. Moderately-soluble compounds (e.g., C_5_H_4_O_2_, furan-2-carbaldehyde) quickly reach their aqueous saturation concentration, and their mixing ratio in the gas phase recovers to their initial steady-state values.

**Fig. 3:**
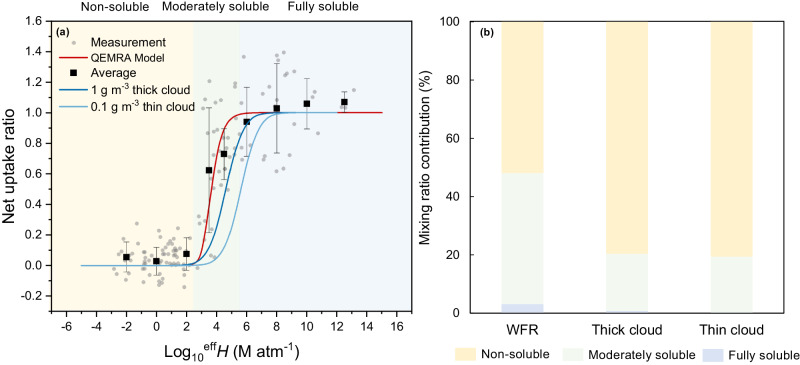
Solubility-dependent uptake ratio for a case study. **a** Representative net uptake ratio (after 3 h of cloud processing) as a function of Henry’s law constants (log_10_ *H*). Grey markers represent uptake ratios calculated for oxidized organic gases measured by the Vocus vs. *H* values of individual molecules calculated by the EPI model (see details in the Supplementary Information). Black squares show the average net uptake ratio with vertical error bars as 1 standard deviation of the measured net uptake ratio. Also shown are the predicted aqueous-phase fractions using the QEMRA model for the measurements (red line, see details in ref. ^23^). **b** Relative mixing ratios of organic gases with different solubility as measured by the Vocus and Dual-EESI for the primary emissions, for the conditions in the WFR, thick cloud and thin cloud. The solubility is classified into three classes: non-soluble gases with log_10_ *H* ≤ 2.5 M atm^−1^, moderately-soluble gases with 2.5 M atm^−1^ < log_10_ *H* ≤ 5.5 M atm^−1^, and fully-soluble gases with log_10_ *H* > 5.5 M atm^−1^ under WFR conditions.

EPI freeware v4.10 (EPI Suite, refer to the Supplementary Information for details on the EPI model) was used to estimate Henry’s law constants using the group contribution and bond contribution methods^29–32^. Figure 3a shows the Henry’s law constants of some species estimated by parameterizing modeled constants from the EPI Suite model (details of the species names are in Supplementary Table 3) and the net uptake ratio, which is the measured uptake ratio normalized by the maximum uptake ratio of 0.54 (due to diffusion limitations of the WFR, see Supplementary Information for details). To describe the partitioning of molecules with different solubility, we employed a kinetic model called QEMRA (see Supplementary Information for details). For organic gases at log_10_ *H* below 2.5 M atm^−1^, the uptake ratio is close to 0. It increases from 2.5 to 5.5 M atm^−1^ and becomes constant above 5.5 M atm^−1^. The uptake coefficients of compounds against these Henry’s law constants follow the same behavior estimated by the QEMRA model, which gives confidence in the parameterized Henry’s law constants and suggests a minor deviation from ideality, expected given the diluted conditions in the water film representative of cloud water. In this work, the pH values of our solutions (pH = 4–5) are similar to those of cloud and fog samples^33–35^. Based on their solubility under our conditions, we categorized the organic gases into three classes: non-soluble gases (log_10_ *H* ≤ 2.5 M atm^−1^), moderately-soluble gases (2.5 M atm^−1^ < log_10_ *H* ≤ 5.5 M atm^−1^), and fully-soluble gases with irreversible uptake (log_10_ *H* > 5.5 M atm^−1^). Estimated Henry’s law constants for all detected wood-burning gaseous species were calculated through a simplified parameterization of detected gaseous species and Supplementary Fig. 7 shows a comparison between EPI suite model log_10_ *H* and parameterized log_10_ *H*, highlighting the general agreement between the two approaches.

By calculating the parameterized log *H* for all organic gases, we estimated that non-soluble and moderately-soluble gases make up 51.9% and 45% of the total mixing ratio of organic gases, respectively under WFR conditions. In our previous WFR study, we demonstrated through parameterization that the liquid water content (LWC) has a significant impact on the aqueous fraction of a given compound^23^. In Fig. 3b, we compare the relative mixing ratios of organic gases with different solubilities under WFR conditions to those in thick and thin clouds (1 g m^−3^ and 0.1 g m^−3^)^36,37^. The definition of the non-soluble, moderately-soluble and soluble gases under thick and thin cloud conditions depends on the curve in Fig. 3a. The fraction of non-soluble gases is higher for thick-cloud and thin-cloud conditions than in the WFR (79.6% and 80.6%, respectively, compared to 51.9%). Overall, 45% of the total NMOG mixing ratio is classified as moderately soluble under WFR conditions, whereas for thick-cloud conditions, it is expected to decrease to 19.3%. This indicates that the fraction of moderately-soluble compounds is to some extent overestimated in the WFR, while still being close to real ambient conditions. To achieve an even more representative simulation of the uptake, a thinner water film would be required. However, under such conditions, the aqSOA composition and yield cannot be determined due to experimental limitations.

### aqSOA composition

The bulk composition of the aqSOA, as determined by the AMS, shows prominent signals of CHO^+^ (*m/z* 29), C_2_H_3_O^+^ (*m/z* 43), and CO_2_^+^ (*m/z* 44), oxygen-containing ions, which typically result from carbonyl and acid functional groups (Supplementary Fig. 8)^10,38,39^. Additionally, previous studies found that methylglyoxal and glyoxal from biomass burning are key precursors globally for secondary organic aerosol (SOA) formation^25,40,41^. Characteristic signals at C_2_H_2_O_2_^+^ (*m/z* 58), C_2_O_2_^+^ (*m/z* 56) and CH_2_O_2_^+^ (*m/z* 46) are observed, which have also been reported in aqSOA derived from methylglyoxal and glyoxal^42,43^. The aqSOA is characterized by a high oxygen-to-carbon (*O/C*) ratio of 0.9 ± 0.1. Such elevated *O/C* ratios for aqSOA are also observed in the field (e.g., San Pietro Capofiume and Beijing), indicating an increase in oxygenation as the aqSOA mass fraction increases^10,44^.

In addition to the bulk characterization by the AMS, the particle-phase EESI-TOF-MS (Particle-EESI) provides detailed molecular information about the aqSOA. We identified an increase in the number of ions above the detection limit with oxidation time. For the pure uptake experiment (without oxidation), 34 ions were detected, whereas, for the 1-hour and 3-hour aqueous phase oxidation experiments, 307 ions and 1275 ions were detected, respectively (Supplementary Fig. 9a). After 3 h, the number of detectable ions remained roughly constant (excess oxidation may lead to fragmentation). To categorize the aqSOA molecular composition, we aggregated the data into three organic classes: CHO, CHN, and CHON. Among these, the CHO class was the largest, constituting 80% of the detected signal (Supplementary Fig. 9b).

The complex oxidation processes can also be illustrated using the carbon oxidation state (OS_C_) and the number of carbon atoms (#C)^45^. Supplementary Fig. 10 shows OS_C_ vs. #C for different oxidation times and demonstrates that the abundance of highly oxygenated molecules with #C > 14 increases substantially with reaction time (from pure uptake to 3 h). The average carbon and oxygen distributions of aqSOA at different reaction times, as depicted in Supplementary Fig. 11, exhibit considerable similarity (after 3 h). The double-bond equivalent (DBE) of aqSOA was calculated based on elemental formulae measured by the EESI-MS. The DBE for the aqSOA did not change any more substantially after 3 h of oxidation (with values between 4.5 and 4.7). These findings suggest that the composition of the aqSOA undergoes minimal changes beyond approximately 3 h of aqueous phase processing (Supplementary Fig. 11). Within the CHO compounds, aqueous-phase oxidation products of three representative phenols from BB were detected in the oxidation samples. The typical oxidation product of guaiacol dimers in the aqueous phase (e.g., C_14_H_14_O_4_, C_14_H_14_O_5_, C_14_H_14_O_6_, C_13_H_12_O_4_, C_13_H_12_O_6_), and oxygenated ring-opening products (e.g., C_6_H_6_O_4_, C_7_H_10_O_6_) were detected after three-hour aqueous processing. These species were also detected in the aqueous-phase oxidation of phenols with hydroxyl radical or triplet excited states of organic carbon in laboratory studies, as well as in cloud water samples and aqSOA from biomass burning in field campaigns^21,44,46–48^.

**Fig. 4:**
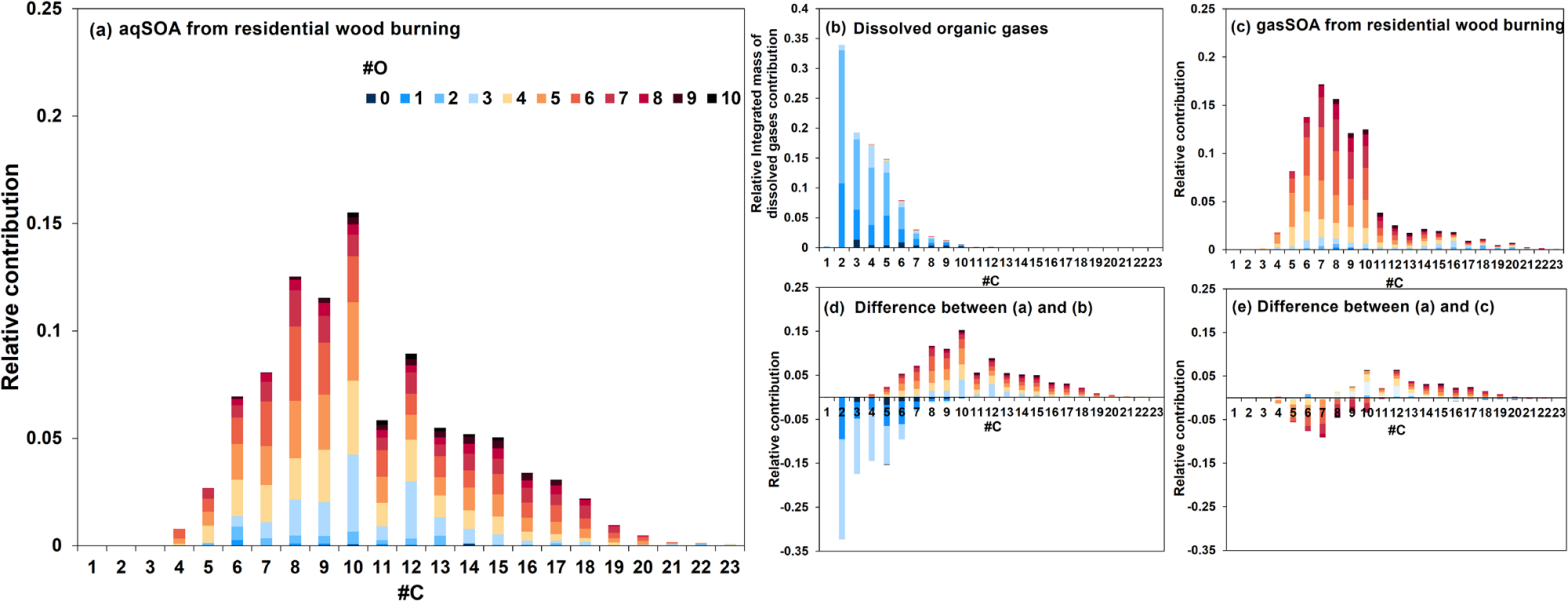
Detailed chemical composition of the aqSOA. The average carbon number distribution (*x*-axis) is colored by oxygen number. **a** AqSOA composition after 5 h of cloud processing measured by the Particle-EESI after nebulization, (**b**) Integrated dissolved gases (determined as the difference in the gas-phase composition with and without the water microfilm) measured by the Vocus and the Dual-EESI and calculated based on experimental data and the QEMRA model result from Fig. 2, (**c**) GasSOA composition from gas-phase oxidation of the same wood burning experiment using an oxidation flow reactor (OFR) measured by the Particle-EESI. The aqueous and equivalent photochemical age for both (**a**) (aqSOA) and (**c**) (gasSOA) are ~5 days. The differences between aqSOA and integrated dissolved gas and gasSOA are shown in **d** and **e**, respectively (positive signal means aqSOA is higher).

Figure 4 displays the molecular composition of aqSOA compared to the gaseous species dissolved in the WFR (determined as the difference in the gas-phase composition with and without the water microfilm). A wide range of ions with carbon numbers ranging from C4 to C23 is observed, with the highest signal attributed to compounds with C8-10 (Fig. 4a). Our findings demonstrate that the composition of aqSOA is significantly more oxygenated and is dominated by molecules containing a greater number of carbon atoms compared to the dissolved gases (Fig. 4b). The number of oxygen atoms (#O) per molecule in aqSOA species, ranging from 3 to 7, is considerably higher compared to the number of oxygen atoms in species dissolved into the WFR water microlayer. The increase in the oxygen content shows that the dissolution of oxidized organic vapors alone cannot explain the aqSOA production in terms of amount and composition. The disconnect between the dissolved gases and observed molecules also provides strong evidence that aqSOA results from the gas-phase water-soluble precursors that partition and react with OH to yield products with a much higher oxidation state, forming aqSOA. The top 50 aqSOA molecules, accounting for approximately 36.7%, consist mainly of oxidized monomers with four to six oxygen atoms. In contrast, the major dissolved gas molecules contain only one to three oxygen atoms.

**Fig. 5:**
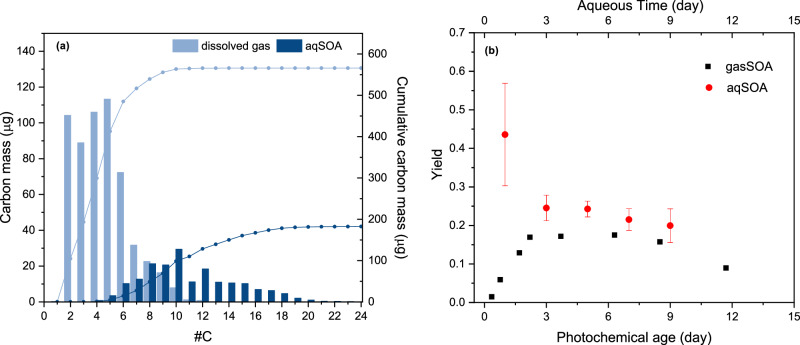
Carbon mass and average SOA yield from aging of biomass burning emissions. **a** Carbon mass distribution and cumulative carbon mass of the oxidation products of aqSOA (light blue bars and light blue line, measured by the Particle-EESI), and of dissolved gases (dark blue bars and dark blue line, calculated based on the gas concentrations measured by the Vocus and the Dual-EESI and calculated from the QEMRA model after 5 h of experiment). **b** Measured aqSOA yields (red circles) compared to yields for SOA from gas phase oxidation (black squares). The aqueous time and photochemical age were calculated based on global average OH concentrations (1.5 × 10^6^ molecules cm^−3^) and typical ambient conditions in cloud droplets. The error bars are the one standard deviation of the aqSOA yield calculated by two labeled isotopes. The yields were calculated as described in the Supplementary Information.

Besides, aqSOA also has a larger contribution from larger carbon-containing species with substantially lower volatility (Supplementary Fig. 12). Homologous series of monomer (C ≤ 5) compounds account for the major dissolved gas fraction (53%), whereas the carbon number per molecule in aqSOA species (C ≥ 8, 85%) is substantially higher than in dissolved gas molecules, indicating that oligomerization plays an important role in the formation of aqSOA (Fig. 5a). By using the nebulization, we cannot exclude that a fraction of the observed oligomers are formed during the aerosol drying process^49^, which also occurs in the ambient atmosphere.

Mass spectral signatures of aqSOA from residential wood burning are characterized by an abundance of highly oxygenated and high molecular weight species. Additionally, we used an oxidation flow reactor (OFR) to investigate gasSOA formation from the same BB emissions after a simulation of approximately five days of photochemical aging^50^. In comparison to aqSOA, the dominant signals in gasSOA are attributed to C_6-8_H_x_O_3-7_ compounds, while more highly oxygenated and carbon-containing species are observed in aqSOA (Fig. 4c), due to extensive oxidation in the gas phase and to oligomerization processes in the aqueous phase.

### SOA yield

The resulting aqueous solutions from different oxidation experiments (1–9 h) were spiked with 5 ppm solutions of isotopically labeled ammonium sulfate (NH_4_)_2_^34^SO_4_ and ammonium nitrate NH_4_^15^NO_3_, and used for quantification of the organic concentration, resulting in several micrograms per cubic meter of OA as measured by the AMS. The normalized integrated aqSOA mass (aqSOA mass divided by total NMOG concentration) increases with the increase of the aqueous phase processing time (Supplementary Fig. 13, Supplementary Table 4). This suggested that the composition of aqSOA is not significantly dependent on the aging time (after 3 h), but the amount of aqSOA produced increases with aging. In this study, we assume the aqSOA yield by the ratio between the integrated aqSOA mass and the integrated mass of all NMOGs (including non-soluble gases) from primary emission that diffused to the WFR wetted wall (see the Supplementary Information). The aqSOA yield decreased from 0.43 (after 1 h) to 0.20 (after 9 h), as shown in Fig. 5b. This trend can be attributed to the solubility of the NMOGs in water, where fully soluble NMOGs continue to contribute to the formation of SOA, while moderately-soluble NMOGs mainly contribute to aqSOA during the initial minutes. Non-soluble NMOGs have limited influence on aqSOA mass (Supplementary Fig. 6). This result will be helpful for SOA modeling and field campaign studies to estimate the formation of aqSOA if only the total NMOGs from biomass burning are available. Besides, using the same method, we also estimated the average yield of gasSOA from wood burning using the OFR, which reached a maximum of approximately 0.17, lower than that of aqSOA.

## Discussion

The large contribution of BB-derived cloud chemistry to aqueous secondary organic aerosol, as highlighted in this study, has important implications for radiative forcing and regional air quality. While our experiment focused on residential wood burning, it is important to acknowledge that aqueous SOA formation will also occur in wildfire plumes, influencing regional climates. By experimentally simulating cloud processing, we demonstrate that the OH-radical-initiated oxidation and oligomerization of biomass burning emissions in the aqueous phase results in significant aqSOA formation, with a yield of 0.2–0.43, higher than the maximum gas-phase SOA yields (0.17). Such low gas-phase SOA yield implies that more than 80% of the oxidation products remain in the gas phase. As these oxidation products are more oxygenated than their precursors, they can potentially form additional aqSOA through their irreversible partitioning and further oxidation in the aqueous phase. The solubility and aqueous phase reactivity of oxidized biomass smoke vapors warrant further future investigations.

To date, some studies have observed that reactions of biomass burning emissions occur in the atmospheric liquid phase from regions including the Po Valley^10,51^ and China^12,52,53^, representing a significant source of SOA mass. However, the detailed aqSOA from field studies is far from enough and the only time aqSOA has been reported with molecular information is during the Chinese haze using an EESI. In this study, Tong et al.^12^ found that under high-NO_x_ and RH conditions aqueous-phase chemistry can contribute substantially to SOA formation, accounting for 53.7% of the total SOA in Beijing. As shown in Supplementary Fig. 14b, the ambient aqSOA in Beijing exhibited a high proportion of O3-6 molecules and an overall enhanced signal from ions with highly oxygenated low carbon ions (e.g., C_6_H_6_O_5_) relative to the other oxygenated organic aerosol. The highest yields of aqSOA were found to be associated with limited atmospheric aging (1 day) and highlight the large aqSOA potential close to emission sources during haze-like conditions with high relative humidity. Although a smaller fraction of soluble gases partitions under fog or haze conditions due to the relatively lower LWC (10^−3^-10^−1^ g m^−3^) compared to clouds^54,55^, these soluble gases will still react in the liquid phase and form aqSOA. However, due to the more complex condition and source for Chinese haze uncertainty compared to our WFR condition (LWC, OH reactivity and potential other chemical pathways)^56,57^, more evidence of aqSOA chemical composition from the field is needed and more haze-relevant experiments should be designed for the lab to do a further comparison.

Extrapolating our results to the other residential wood-burning experiments we did using an OFR or smog chamber, we estimate that the contribution of aqSOA is 2–7 times higher than the contribution of primary biomass-burning organic aerosol (BBOA) emissions, depending on the emission factors of the primary organic gases and BBOA. This result is similar to the ambient atmosphere during haze formation, with the contribution of aqSOA surpassing that of BBOA by a factor of 2–4^12,52,53^. Our simulation experiments suggest that aqueous processing of biomass burning vapors can be one of the important sources of observed aqSOA during haze conditions.

In addition to residential wood burning, it is crucial to note that wildfires or prescribed open burning have become more frequent in many regions due to heatwaves and droughts^58,59^. Expanding upon our experiment’s focus on organic gases from primary residential wood burning, the WE-CAN aircraft campaign systematically characterized the western U.S. wildfire plumes and found that wildfires are also a major source of VOCs to the atmosphere, emitting hundreds to thousands of different organic gas-phase species^60,61^. Some of these organic gas-phase species will not only react with OH radicals in the gas phase but will also dissolve in cloud water, contributing to aqSOA formation. Therefore, further research is needed to investigate the solubility of organic vapors from wildfires and their resulting aqueous SOA yield.

In the atmosphere, organic gases from photochemical reactions in the ambient atmosphere are more oxidized than primary emissions. A higher oxidation state makes them more water soluble and they can partition even more than primary products into the aqueous phase, where their reactions can form additional aqSOA mass under higher RH conditions during the day, and primary emissions from biomass burning are the major aqSOA precursors at night^62,63^. Additionally, Wang et al.^63^ found that gasSOA could also be transformed to aqSOA during daytime under higher NO_x_. Therefore, our results represent the first step toward a better representation of in-cloud aqSOA formation from residential wood burning while additional experiments beyond the conditions presented here are needed to investigate aqSOA formation (e.g., in the presence of extra UVB light, varied pH, NO_x_ and temperature).

## Methods

### Wetted-wall flow reactor

An experimental simulation of aqSOA formation from residential wood burning emissions was conducted using a wetted-wall flow reactor (WFR) and a suite of mass spectrometers. In this study, an in-house built WFR operating in continuous flow mode was used to simulate the in-cloud formation and aging process^23^. The WFR consists of a quartz glass cylinder (length, 125 cm; internal diameter, 6 cm), a Xe-excimer laser, a humidifier, and a water injection system. The inner surface is sandblasted to increase the wettability of the glass. The cylinder rotates at a speed of 15 rotations per minute, maintaining an 85 µm water microlayer on the wall. A stirring bar (length = 123 cm, diameter = 3 mm) was placed at the bottom of the cylinder, facilitating the wetting of the wall and maintaining the microfilm. The Xe-excimer laser (7.2 eV, 172 nm) is used to induce photolysis of H_2_O and O_2_, resulting in the formation of OH radicals as well as O_3_ and HO_2_ molecules. All experiments were conducted with a stream of synthetic clean air (9.5–9.75 L min^−1^) generated by a zero air generator. This dry air stream was passed through a porous PTFE Gore-Tex tube (Gore™) immersed in ultrapure water to keep a constant RH of 95%–100%. The temperature of the WFR was measured with two thermocouples of type K, with one positioned at the inlet and the second at the outlet. RH was monitored with a hydroclip (Rotronic HygroClip). At the end of the WFR, there is a water injection system, which consists of a tube with one side in the quartz glass cylinder and another side connected to the injector. After each experiment, the WFR was cleaned three times by rotating it for 30 min with 500 mL of ultrapure water (18 MΩ cm). Subsequently, the WFR walls were then heated using a heat gun to 200 °C, while a pure dry air stream of 20 L min^−1^ was maintained for 1 h. Despite the higher LWC (6 × 10^−3^ g mL^−1^) in the WFR compared to a cloud, our non-equilibrium system reflects well what will happen in the atmosphere.

### Experimental setup

The experimental design is shown in Supplementary Fig. 2. Before each burn and throughout the WFR experiments, a continuous stream of pure air was passed through the gas lines overnight to prevent cross-contamination between burns and to ensure a low background of VOCs. To simulate residential wood combustion, pine and spruce wood logs from a local forestry company in Würenlingen, Switzerland, weighing between 0.5 and 1.2 kg, were combusted in a modern woodstove (Avant, 2009, Attika). Emissions were sampled from the chimney through a heated line (473 K), diluted by a factor of approximately 10 using an ejector diluter (453 K, DI-1000, Dekati Ltd.) and injected into a holding tank (stainless steel, ~1 m^3^) through a heated stainless-steel line (423 K). The holding tank ensured a constant concentration of NMOGs needed as input for the WFR experiments. The ejection diluter was operated with an input pressure of ~1.5 bar and the singular injecting time to the holding tank was 10–20 min. During the injection of the emissions into the holding tank, the concentrations of carbon monoxide (CO), carbon dioxide (CO_2_), total hydrocarbon (THC), NO_x_ and particles were measured in situ (see Supplementary Table 1). In most of the experiments, the modified combustion efficiency (MCE, defined as ΔCO_2_ / (ΔCO + ΔCO_2_)) ranged from 0.9 to 0.95, indicating a flaming burning phase slightly mixed with a smoldering burning phase. Once the emission concentration in the holding tank reached a considerable level (THC concentrations > 15 ppmv C), the injection was halted and the organic gases from the holding tank were introduced into the WFR after passing through a particle filter (quartz) and mixing with the humidified air stream. The SVOC contribution in the gas phase is low^64^ and the SVOCs in the particle phase are filtered and not considered in this study.

Experiments were performed at approximately 100% RH and a temperature of 290–295 K in the WFR. The humidified air stream was measured as the background. In short, this study comprises two distinct types of experiments. One is a pure uptake experiment (blank experiment), conducted with the Xe-excimer laser turned off and no generation of OH radicals. The first phase of this experiment was carried out with the Xe-excimer laser deactivated, meaning no oxidants were introduced, and it was referred to as the ‘primary emissions’ phase. Subsequently, with the Xe-excimer laser still deactivated, 20 mL water was injected into the WFR and rotated (15 rotations min^−1^) to maintain a water microlayer on the wall. After 1 h, the water mixture was collected and analyzed. This experiment is used to verify if the vapor uptake, in the absence of oxidants, leads to the formation of aerosol upon nebulization. The second type involves oxidation experiments lasting 1, 3, 5, 7, and 9 h. Initially, like the blank experiment, organic vapors were continuously injected from the holding tank into the WFR (‘primary emission’ phase with Xe-excimer off). Subsequently, the Xe-excimer laser was turned on to create the oxidants, denoted the ‘oxidation’ phase. The OH concentrations at steady-state conditions in the WFR were 3 × 10^−13^ to 5 × 10^−12^ M in the aqueous phase and 3 × 10^8^ molecules cm^−3^ in the gas phase. Once the gas concentration in the WFR reached stability (approximately 15 min), 20 mL water was injected and rotated, while the Xe-excimer laser was turned on until the end of the experiment. At this point, the water mixture was collected and stored at 4 °C until chemical analysis, which was analyzed by AMS and EESI within 24 h.

Lee et al.^65^, observed the direct production of hydrogen peroxide and formaldehyde from biomass burning, and these species from biomass burning will contribute to odd-hydrogen radical production, thereby affecting the oxidizing capacity of the atmosphere. However, the results from the blank experiment showed that extremely few organic compounds dissolved into the water and formed aqSOA (Supplementary Figs. 9, 10), suggesting a negligible impact of these compounds compared to the SOA formation in the presence of OH radicals.

### Measurements

A dual-phase extractive electrospray ionization time-of-flight mass spectrometer (Dual-EESI) and a PTR-TOF-MS (PTR-MS for short, Ionicon, Series 8000) were used for the chemical characterization of the primary gas-phase compounds and their oxidation products emitted from the wood burning. In a subset of experiments, a Vocus PTR-TOF-MS (Vocus for short, TOFWERK, Aerodyne, Inc.) was also deployed to get a broader spectrum of VOCs, IVOCs and their oxygenated products (compared to the conventional PTR-MS). These additional measurements were used to determine the uptake ratio of gaseous products and the Henry’s law constant of individual molecules for the development of parameterization. Ozone concentrations were monitored using a commercial monitor (Monitor LabsInc., 8810). The uptake ratio was calculated as (*C*_no-cloud_-*C*_cloud_)/*C*_no-cloud_, where *C*_no-cloud_ represents the concentration of organic gases after oxidation and *C*_cloud_ represents the concentration of organic gases after e.g., 3 h of cloud simulation.

Offline analysis of aqueous-phase chemical composition was explored using two different instruments. A portion of the resulting aqueous solutions was nebulized in N_2_, using a customized Apex Q nebulizer (Elemental Scientific Inc., Omaha, USA) operating at 60 °C. The resulting droplets were dried using a Nafion dryer and analyzed using a high-resolution time-of-flight aerosol mass spectrometer (AMS, Aerodyne) and a Particle-EESI.

### Supplementary information

Supplementary Information

## Data Availability

The data used in this work are archived and can be downloaded from Zenodo (10.5281/zenodo.10630474).
