# Supplementary material for: Large contribution of in-cloud production of secondary organic aerosol from biomass burning emissions
Source: NPJ Clim Atmos Sci. 2024 Jun 25;7(1):149. doi: 10.1038/s41612-024-00682-6 (PMC11199137; doi:10.1038/s41612-024-00682-6)
Supplement: Supplementary file 1 — Supplementary Information [file 41612_2024_682_MOESM1_ESM.pdf]

Supplementary Information for

Large contribution of in-cloud production of secondary organic aerosol from biomass burning emissions

Tiantian Wang, Kun Li[a], David M. Bell, Jun Zhang, Tianqu Cui, Mihnea Surdu, Urs Baltensperger, Jay G. Slowik, Houssni Lamkaddam*, Imad El Haddad*, Andre S. H. Prevot*

PSI Center for Energy and Environmental Sciences, Paul Scherrer Institute, 5232, Villigen, Switzerland

[a]now at: Environmental Research Institute, Shandong University, Qingdao, 266237, China

* Corresponding author(s). E-mail(s): andre.prevot@psi.ch; houssni.lamkaddam@gmail.com; imad.el-haddad@psi.ch

This file includes:

Supplementary notes 1 to 3

Supplementary Figures 1 to 14

Supplementary Tables 1 to 4

Supplementary References

Supplementary notes 1. Operation and calibration of the instruments

PTR-TOF-MS

A PTR-TOF-MS 8000 (Ionicon Analytik GmbH, Innsbruck, Austria) was deployed for measuring organic gases. The detailed principle and operation of the PTR-TOF-MS are described thoroughly elsewhere (PTR-MS for short)[1, 2]. The PTR-MS was operated using protonated water (H_3O^+) as the reagent ion and measures non-methane organic gases (NMOGs) having a proton affinity greater than that of water. The operating pressure, temperature and voltage in the drift tube were held at 2.2-2.3 mbar, 60 °C and 600 V, respectively, which resulted in a reduced electric field (*E/N*) value of ~130 Td. Calibrations were performed twice (before and after the entire measurement campaign) by a 13-compound gas standard cylinder

(Tofwerk AG) including carbonyls, alcohols, halogens and aromatics from *m/z* 42 to *m/z* 137 (1000 ppbv each). The inlet consisted of polyether ether ketone (PEEK) tubing (outer diameter o.d. = 3 mm) and the sampling line was heated (333 K) with a flow rate of 0.2 L min^{-1}, and the time resolution was set to 60 s. The raw data were processed using the Tofware post-processing software (Tofware version 2.5.11, Tofwerk AG, Thun, Switzerland). A detailed description of the data analysis protocol can be found in Wang et al. [3, 4]. The volume mixing ratios in ppbv were calculated based on the method proposed by de Gouw and Warneke [5] and applying the literature reaction rates (*k*) of the ion with the H_3O^+ ion when available [6]. For ions without a known reaction rate, a rate constant of 2×10^{-9} cm^3 s^{-1} was assumed.

Vocus-PTR-TOF-MS

For some experiments, a Vocus PTR-TOF (Tofwerk; hereinafter referred to as Vocus) was deployed in addition to the PTR-MS to measure organic vapors with a wider range of volatilities. A detailed description of the Vocus is provided elsewhere [7]. Compared to the conventional PTR-MS, it has greatly enhanced sensitivity due to a newly designed chemical ionization source, and it can detect a broader spectrum of volatile organic compounds (VOCs), intermediate-volatility organic compounds (IVOCs), and their oxygenated products (up to six to eight oxygen atoms for monoterpene oxidation products). For this study, the Vocus was operated with H_3O^+ as the reagent ion. Sample air was drawn in through a 1 m long polytetrafluoroethylene (PTFE) tube (6 mm o.d.) using a total sample flow of 4.3 L min^{-1}, which helped to reduce inlet wall losses and sampling delay. Of the total sample flow, only 100-150 cm^3 min^{-1} went into the Vocus and the rest was exhausted. The Vocus was calibrated before and after the measurement every day using a multi-component standard cylinder (Tofwerk AG). The standard gases were diluted by the injection of zero air, producing VOCs mixing ratios of around 20 ppbv. The calibration components were methanol, acetaldehyde, acetonitrile, acetone, acrylonitrile, isoprene, methyl ethyl ketone, benzene, toluene, m-xylene, α-pinene, and 1,2,4-trimethylbenzene. The background measurements were performed using dry zero air every day. Data were recorded with a time resolution of 1 s. The raw data were processed using the Tofware v3.2.3 software (TOFWERK, Aerodyne, Inc.). The standard non-targeted analysis workflow developed by Tofwerk was adopted for mass calibration and peak fitting. The mass transmission function and the ratios between the measured and calculated sensitivities for a series of ions were used to quantify the data and convert the ion counts to ppbv. A rate constant of 2×10^{-9} cm^3 $molecules^{-1}s^{-1}$ was assumed. Peaks were identified in the range of 40-250 *m/z* and included mostly VOCs and IVOCs

EESI-TOF-MS

The extractive electrospray ionization time-of-flight mass spectrometer (EESI-TOF-MS) provides online, highly time-resolved measurements of gas- and/or particle-phase organic molecular ions without thermal decomposition or ionization-induced fragmentation. In this study, two configurations of the EESI-TOF-MS were used, for online gas-phase and offline particle-phase measurements, respectively. The particle-phase EESI-TOF-MS (Particle-EESI for short) is described in detail elsewhere [8]. For the gas phase, a dual-phase extractive electrospray ionization time-of-flight mass spectrometer (Dual-EESI for short) configuration was adopted [9]. The systems differ only in the particle/gas-handling front end installed upstream of the ionization zone. For the online Particle-EESI measurement, the aerosol was first passed through a denuder to eliminate the gaseous fraction. The resulting flow was then alternately sampled directly or through a particle filter, which yields the instrument background. However, in this study, the offline filter measurements utilized nebulized MilliQ water rather than interposing a particle filter and the difference of these two measurements yielded the particle composition. For the dual-phase measurements, emissions from wood burning were alternately sampled directly or through a particle filter, whcih yielded the gas-phase composition.

For both instruments, the sample flow intersected a charged spray of 1:1 water: acetonitrile doped with 100 ppm NaI (99 %, Sigma-Aldrich), and the mass spectrometer was configured to detect positive ions. Analyte ions were detected almost exclusively as $[M]Na^+$, with the Na^+ charge carrier mostly suppressing other ionization pathways. Data analysis, including high-resolution peak fitting, was performed using Tofware version 2.5.7 (Tofwerk AG, Thun, Switzerland). A description of the detailed data treatment processes can be found elsewhere [10, 11]. Previous tests of the Dual-EESI showed that it is sensitive to the change of the water liquid content in the WFR and we could not measure the time series after the water injection. In this study, only the primary emission and oxidation products from residential wood burning were measured by the Dual-EESI. Quantification of the Dual-EESI measurements by instruments is challenging because the instrument sensitivity varies strongly with molecular identity. As illustrated in Supplementary Figure 3, compounds sharing the same molecular formula with the Dual-EESI and the Vocus were used to quantify the Dual-EESI data. Note that this assumes that both instruments measure the same distribution of isomers for ions having a given molecular formula.

AMS

The non-refractory particle composition was monitored by a high-resolution time-of-flight aerosol mass spectrometer (HR-TOF-AMS, Aerodyne Research Inc.). The offline AMS operation and AMS analysis were similar to other AMS measurements [12]. Data analysis started with raw AMS data processed through SQUIRREL (SeQUential Igor data RetRiEvaL v. 1.63; D. Sueper, University of Colorado, Boulder, CO, USA) and PIKA (Peak Integration and Key Analysis v. 1.23) to obtain mass spectra of identified ions over the *m/z* range 12-120.

Holding tank

As shown in Supplementary Figure 2, the biomass-burning emissions were introduced into the holding tank through heated lines with the heated ejection diluter. The holding tank ensured a constant concentration of NMOGs needed as input for the WFR experiments. The composition in the holding tank is stable and all the gas-phase species in the WFR are from the holding tank. Compared to the “smog chamber” the wall losses were much less due to the much higher concentration (more details could be found in Li et al. (2024)). We have also done the test to measure the decay of VOC and IVOC concentrations in the holding tank, which is 10% in 5-6 h for either VOCs or IVOCs and mostly comes from dilution rather than wall losses. This has been corrected for dissolved gas calculation. We have also evaluated the losses of IVOCs and VOCs in the holding tank and between the holding tank and the WFR inlet (after the pump) and found negligible losses.

Supplementary notes 2. Calculations

Volatility and water solubility parameterization of the oxidation products

The volatility (i.e. the saturation mass concentration, *C**) for individual organic compounds was calculated based on the number of oxygen, carbon, and nitrogen atoms in the compound using the approach by Li et al. [13]:

$$\log_{10} C^* = (n_C^0 - n_C^i)b_C - n_O^i b_O - 2\frac{n_C^i n_O^i}{n_C^i + n_O^i} b_{CO} - n_N^i b_N \quad (1)$$

where n_C^0 is the reference carbon number; n_C^i, n_O^i and n_N^i denote the numbers of carbon, oxygen and nitrogen, respectively in the compound. b_C, b_O and b_N are the contributions of each atom to $\log_{10} C^*$, respectively; and b_{CO} is the carbon-oxygen non-ideality [14]. The parameters used in this analysis are presented in Supplementary Table 2.

Measured ions are separated into volatility classes as described in the main text. For the Dual-EESI measurements, 68.9% of the measured NMOG mixing ratio are VOCs and 31% are IVOCs, while the remaining 0.1% are semi-volatile organic compounds (SVOCs) and low-volatility organic compounds (LVOCs). For the PTR-MS, around 2% of the measured NMOGs are IVOCs, and 98% are VOCs. In contrast, the fraction of IVOCs measured by the Vocus is 14.5%, indicating that most of these IVOCs are not fully quantified with the PTR-MS (Supplementary Figure 4). We also conducted other burning experiments by directly sampling the smoke in real-time characterization of gas-phase emissions during various burning phases using Vocus. The results show that the SIVOC contribution is less than 0.4% and the IVOC contribution is 11.9 ± 3%, which is aligned with the 14.5% in this study. Compared to conventional PTR-MS instruments, the observation of larger hydrocarbon molecules by the Vocus is mainly caused by the much lower wall losses and increased detection efficiency. Organics with the lowest oxidation extent were better observed by the Vocus than the Dual-EESI, while organics with the largest molecular weights and highest oxidation extent were better observed by the Dual-EESI.

Previous studies showed a strong negative correlation between Henry's law constants and C^* [15, 16]. In our study, multiple regression was used and p-values of less than 0.05 mean the parameters were statistically significant. Estimated Henry's law constants for all wood-burning oxidation products were calculated through a simplified parameterization of the water solubility as a function of volatility and oxygen-to-carbon (*O/C*) ratio by the following equation:

$$\log_{10} H = 3.6 * \frac{O}{C} - 1.37 * \log_{10} C^* \qquad (2)$$

Supplementary Figure 7 depicts a good agreement between the two data sets.

EPI model and QEMRA model

Estimation Programs Interface (EPI) Suite is a widely used software developed by the US Environmental Protection Agency to predict the physicochemical properties of organic compounds. Here, based on the structural information of the species from biomass burning literature, Henry's law constants were calculated. The structural input for the calculation was the SMILES (Simplified Molecular Input Line Entry System) code, which is also used by other estimation programs in EPA's EPI Suite.

In the current work the following subprograms of EPI Suite v4.10 were employed: Henrywin v3.20 estimates Henry's law constants at 25 °C through the group contribution and bond contribution methods, (available on http://www.epa.gov/oppt/exposure/pubs/episuite.htm). Supplementary Table 3 shows the potential name of the compounds detected and the calculated Henry's law constants by EPI.

The QEMRA model (aQuEous-hydroMeteor phase pRocessing and mAss transfer model) is a kinetic model, which was used to estimate the fraction of water-soluble compounds taken up into the WFR water microfilm and the subsequent aqueous OH-oxidation in previous WFR studies (more details are founded in Lamkaddam et al.[16]). Due to diffusion limitations of WFR, also fully soluble compounds can only partially pass through the system and do not touch the aqueous layer, indicating that 54% of the reaction mixture diffuses to the surface of the water microlayer. Therefore, in Figure 3a, the net uptake ratio is normalized by 0.54. The QEMRA model is also used for the estimation of dissolved gases that could only be measured by the Dual-EESI (Figure 3b, Figure 4a, and Supplementary Figure 13). As mentioned above, the compounds measured by the Dual-EESI are only available for "primary emission" and "oxidation" stage. Based on the volatility, we could estimate the solubility and uptake ratio and then calculate how much gases are dissolved in the water.

In opposition to our kinetically limited system, in the atmosphere, the partitioning between both phases happens at quasi-equilibrium and is represented by the following equation:

$$f_{aq} = \frac{LWC/C_{aq}^{*}}{1+LWC/C_{aq}^{*}} \quad (3)$$

where f_{aq} is the equilibrium fraction in the aqueous phase, LWC is the liquid water content (in µg m^{-3}), and C_{aq}^{*} is the saturation vapor concentration over water (in µg m^{-3}) and is equal to 10^{12} $H^{-1}R^{-1}T^{-1}$ (where R = 0.0821 L atm mol^{-1}K^{-1} and T is the temperature in K). The organic aqueous fraction through the dissolution of the gas phase into the WFR water microfilm can be assessed by monitoring its variation with the QEMRA model. This equation was used to calculate the solubility under thick and thin cloud (Figure 3a).

Aqueous secondary organic aerosol (aqSOA) mass assessment and yield

The total amount of the collected aqueous samples (V_{liquid}) was determined by weighing. These samples were then spiked with 5 ppm solutions of isotopically labeled ammonium sulfate $(NH_4)_2{}^{34}SO_4$ and ammonium nitrate $NH_4{}^{15}NO_3$ (Sigma Aldrich, purity >99%), nebulized, and

dried, resulting in an aerosol containing several micrograms per cubic meter of OA, as measured by the AMS (the volume of the isotopically-labeled solution depends on the resulting solution collected from the WFR). This OA concentration was then used to determine the aqSOA in the aqueous phase (in g mL^{-1}) by using the ^{15}N and ^{34}S concentrations measured by the AMS. The mass of organics in the aqueous solutions was calculated using:

$$M_{\text{Integrated SOA, liquid}} = \frac{C_{\text{isotope, liquid}} \times C_{\text{organics, AMS}}}{C_{\text{isotope, AMS}}} \times V_{\text{liquid}} \quad (4)$$

Where $C_{\text{isotope,liquid}}$ is the isotope concentration (ppmv), which was added to the samples; $C_{\text{organics, AMS}}$ and $C_{\text{isotope, AMS}}$ are the organics and isotope concentrations, respectively measured by the AMS. V_{liquid} is the volume of the collected water mixture from the WFR. Then the mass of aqSOA was normalized to the total NMOG concentration for the respective burning experiment:

$$M_{\text{Normalized Integrated SOA,liquid}} = \frac{M_{\text{Integrated SOA,liquid}}}{C_{\text{NMOGs}}} = \frac{M_{\text{Integrated SOA,liquid}}}{C_{\text{VOCs}} + C_{\text{IVOCs}}} \quad (5)$$

The gas-phase organic compounds were measured with the PTR-MS for all experiments. For a subset of experiments, a Vocus and Dual-EESI were deployed to measure organic vapors with a wider range of volatilities (compared to a conventional PTR-MS). Therefore, the total concentration of NMOGs was calculated as the sum of the VOC concentration measured by the PTR-MS (C_{VOCs}) and the IVOC concentration (C_{IVOCs}) An average molecular weight of 125.1 Da and an average IVOCs contribution of 15.7% to the total organic gases concentration was determined based on the molecular formulae and their concentrations, which are measured by the Dual-EESI and the Vocus. In this study, the yield calculation is based on all primary organic gas concentrations, including non-soluble gases as follows:

$$Yield = \frac{Mass_{\text{SOA}}}{Mass_{\text{Organic gases}}} = \frac{M_{\text{Integrated SOA,liquid}}}{0.54 * Flow * \Delta t * Mass\ concentration_{\text{Organic gases}}} = \frac{M_{\text{Integrated SOA,liquid}}}{0.54 * Flow * \Delta t * \left(\sum \frac{C_{\text{VOCs}} * M}{24} + \sum \frac{C_{\text{IVOCs}} * M}{24}\right)} \quad (6)$$

Calculation of photochemical age and aqueous processing time

The OH exposure in the OFR was estimated by the decay of benzene and toluene as measured with the PTR-MS. The equivalent photochemical age (PCA) was calculated using a global average OH concentration of 1.5 × 10^{6} molecules cm^{-3} [17]:

$$PCA = \frac{1}{[OH](K_{\text{X}} - K_E)} \times \left[ln \frac{[X]}{[E]} \Big|_{\text{t=0}} - ln \frac{[X]}{[E]} \right] \quad (7)$$

Where $[OH]$ is the concentration of hydroxyl radicals (OH); K_X and K_E are the rate constants between OH radicals and benzene and toluene, respectively, and $ln\frac{[X]}{[E]}|_{t=0}$ and $ln\frac{[X]}{[E]}$ are the initial emission and measured ratios between benzene and toluene, respectively. For the OFR, the O_3 concentration is 4 ppmv and the OH concentration that is equivalent to ~5 days of aging (shown in Figure 5) is 5.4×10^{11} molecules cm^{-3}. More details of OFR could be found in another paper about gasSOA from biomass burning [18].
The gas-phase OH exposure in the wetted-wall flow reactor (WFR) was estimated by measuring the decay of benzene and toluene using the Vocus instrument, which was connected to the end of the WFR. The decay can be approximated by a linear decay, and the average decrease of benzene and toluene in the WFR represents approximately half of the decrease at the end of the WFR (~ 0.9 hours).

The OH concentration in the WFR is ~3×10^8 molecules cm^{-3} and the O_3 concentration in this condition is between 300 and 350 ppbv. OH radicals, which were continuously injected, are much more soluble and reactive than ozone. Ozone is very selective to compounds with double bonds. The initial OH radical concentration from the excimer light is 1×10^{11} molec cm^{-3} and the initial HO_2 radical concentration is estimated to be two times higher based on previous research [16, 19]. HO_2 radicals were produced with OH radicals from the Xe-excimer laser. The HO_2/OH ratio estimated in this study is consistent with previous cloud studies [20, 21, 22], faithfully reproducing oxidation conditions in the atmosphere. Therefore, we expect HO_2 to play a similar role in the WFR and the atmosphere. Based on our previous WFR experiment with the same set-up, this yields an OH concentration of 3×10^{-13} to 5×10^{-12} M in the aqueous phase, which compares well to typical ambient conditions in cloud droplets [10^{-14} to 10^{-12} M] [23]. Therefore, the aqueous processing time in this study was calculated as follows:

$$Aqueous\ time = \frac{WFR\ oxidation\ time}{[OH]_{\text{ambient cloud droplets}}} \times [OH]_{\text{WFR}} \qquad (8)$$

Supplementary notes 3. Beijing field campaign

The measurements took place from late October to mid-December 2017 in urban Beijing and several haze events were observed. The source apportionment analysis yielded four secondary factors including more-oxygenated aerosol from aqueous-phase chemistry (MO-OOA_{aq}). One of the major haze events was characterized by a high aerosol liquid water content, yielding MO-OOA_{aq} concentrations corresponding to 40.8% of the total OA [24]. Some differences were

observed when we compared the aqSOA composition in this study and aqSOA from the Beijing campaign, such as a relatively higher carbon distribution (C9-C12) and oxygen distribution (O2-O3) in ambient aqSOA compared to laboratory-generated aqSOA. These differences can be attributed to suboptimal operation conditions in Beijing, including denuder breakthrough and high background signals, which significantly increased uncertainties in the data from the EESI-TOF. As a result, a volatility-based filter was applied to select ions for source apportionment, leading to an underestimation of contributions from lower carbon number ions and highly oxygenated ions in Beijing.

Supplementary Figures and Tables:

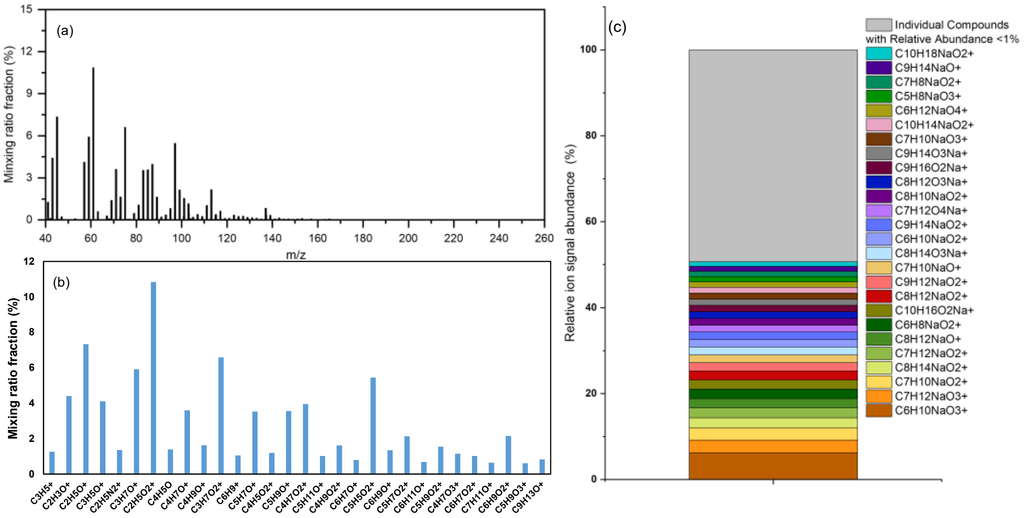

Supplementary Figure 1. (a) High-resolution mass spectra of primary wood burning emissions measured by the Vocus; (b) Mixing ratio fractions (%) of the most abundant species in the Vocus spectra, ordered by molecular weight; (c) Relative ion signal abundance of the gas-phase compounds having a relative abundance of >1% detected during the 26 controlled stack burns using the Dual-EESI. The gray shading denotes the total of compounds having individual relative abundances of <1%.

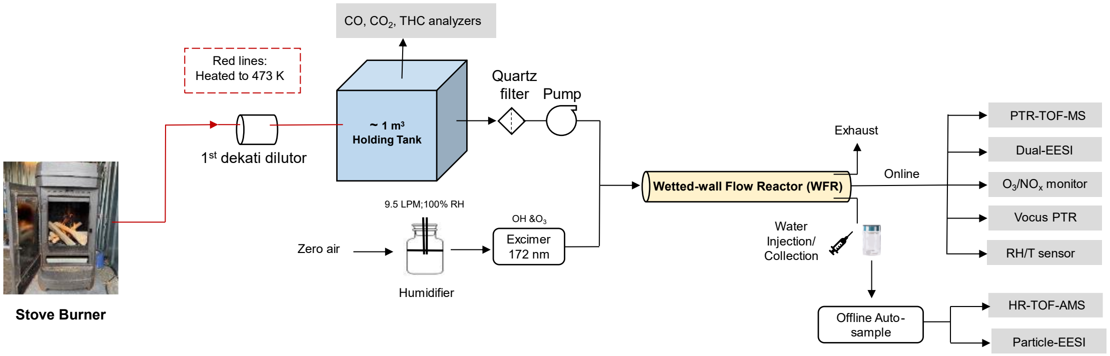

Supplementary Figure 2. Overview of the experimental system, including holding tank (blue shading), WFR (yellow shading), and measuring instruments (gray shading).

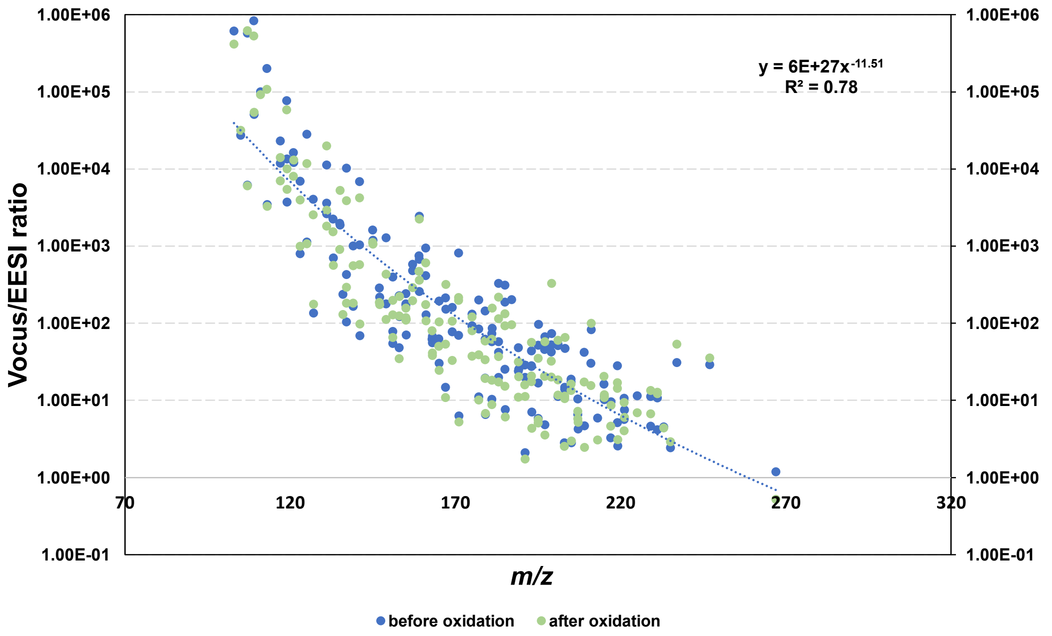

Supplementary Figure 3. Vocus to Dual-EESI concentration ratio for commonly identified molecular formulae as a function of mass-to-charge ratio *m/z*.

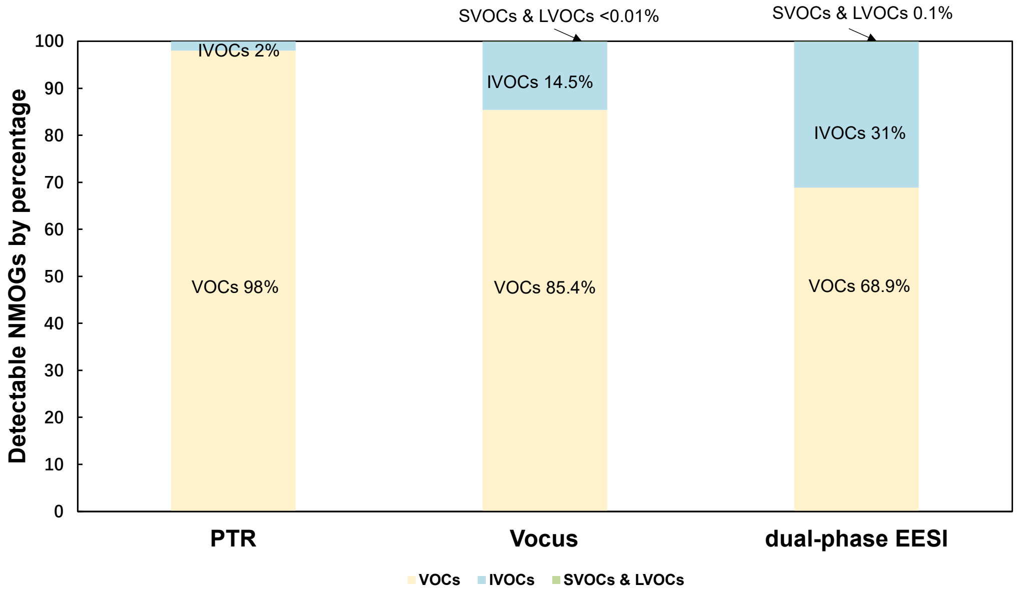

Supplementary Figure 4. Contributions of VOCs, IVOCs, SVOCs and LVOCs to NMOGs were measured with the PTR-MS, the Vocus and the Dual-EESI (data is from a 9-hour oxidation experiment).

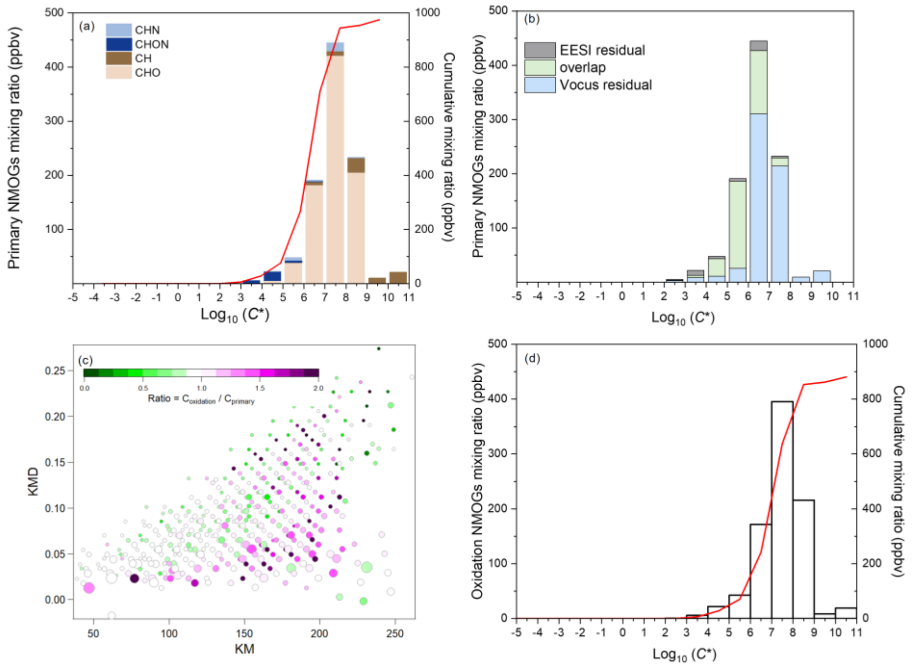

Supplementary Figure 5. (a) Volatility distribution of primary emissions as a function of binned saturation vapor concentration. Columns are color-coded by ion family (CH, CHO, CHN, CHON); (b) Volatility distribution of primary emissions as a function of binned saturation vapor concentration. Columns are color-coded by different instruments; (c) Mass defect plot of the ions identified by high-resolution analysis of the Vocus data set. The dot colors represent the ratios of the mixing ratios after and before oxidation (pink colors indicate higher concentration after oxidation than in primary emissions, see equation in the figure) and the size represents the *O/C* ratio; (d) Volatility distribution of emissions after oxidation as a function of binned saturation vapor concentration.

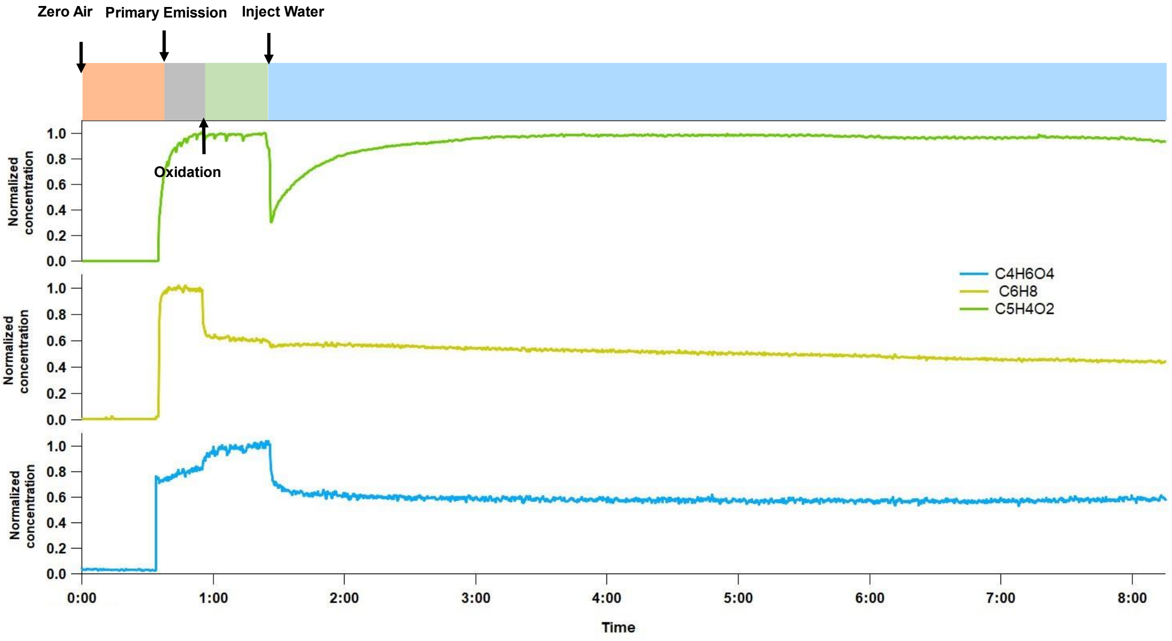

Supplementary Figure 6. Time series of representative species with different solubility. The experiment consists of four phases: zero air (orange): background measurement with zero air and water vapor. Primary emissions (grey): Injection of primary gases from residential wood burning from the holding tank mixed with the humidified air stream. Oxidation (green): initiation of oxidation by switching on the Xe-excimer lamps resulting in the

production of OH radicals. Inject water (blue): Injection of the water microfilm. The normalized concentration represents each concentration value in the entire time series divided by the highest concentration value in that time series.

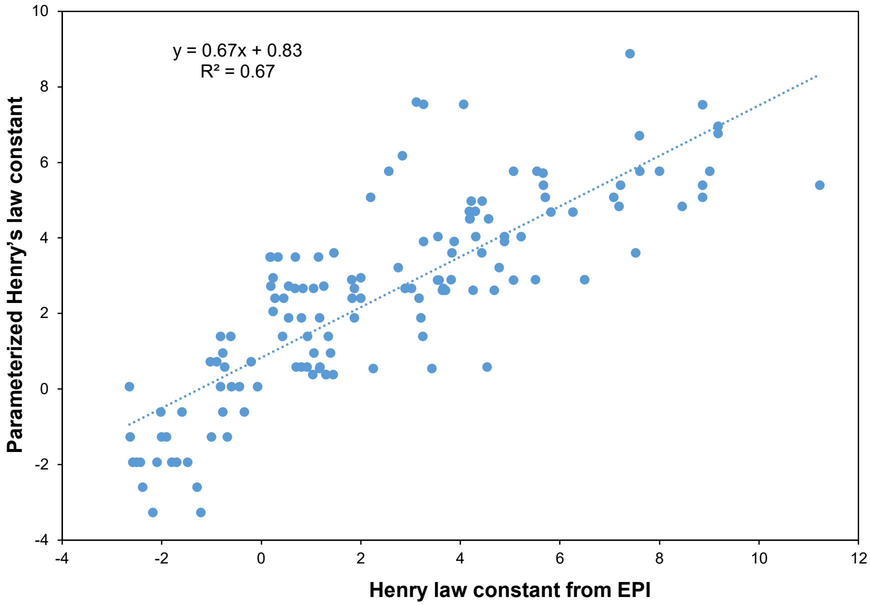

Supplementary Figure 7. Parameterized Henry's law constants ($\log_{10} H$ M atm^{-1}, x-axis) as a function of Henry's law constants from EPI ($\log_{10} H$ M atm^{-1}, y-axis).

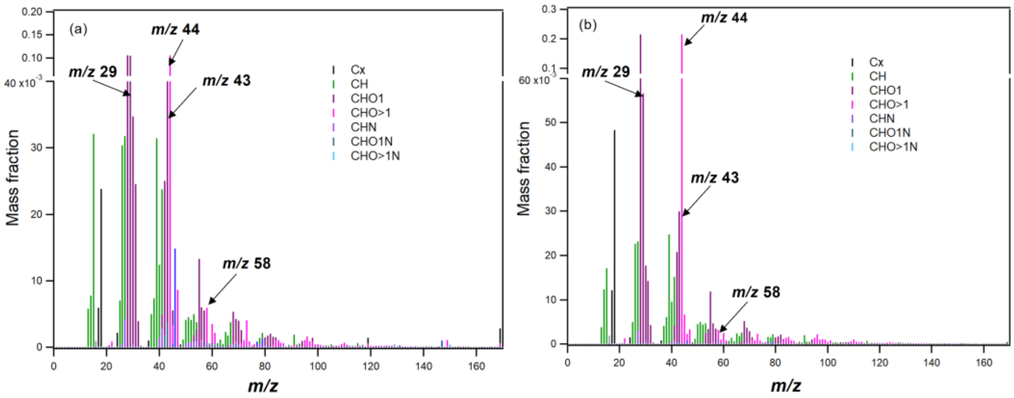

Supplementary Figure 8. Representative AMS spectrum of aqSOA (a) uptake experiment (without oxidation) and (b) oxidation experiment.

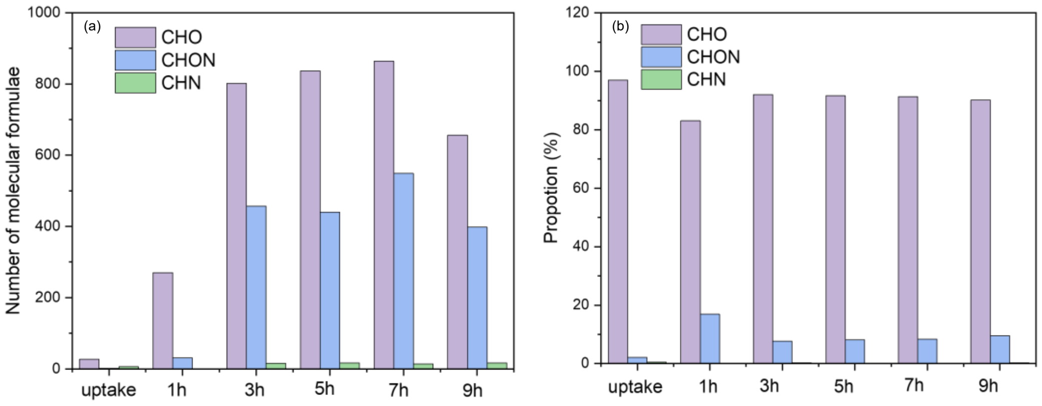

Supplementary Figure 9. (a) Number of identified molecular formulae in aqSOA (measured with Particle-EESI) as a function of reaction time, colored by chemical family (CHO, CHON, CHN). Due to the long time of the experiment (9 hours), excess oxidation may lead to fragmentation. (b) Fractions of molecular formulae corresponding to a given family as a function of reaction time.

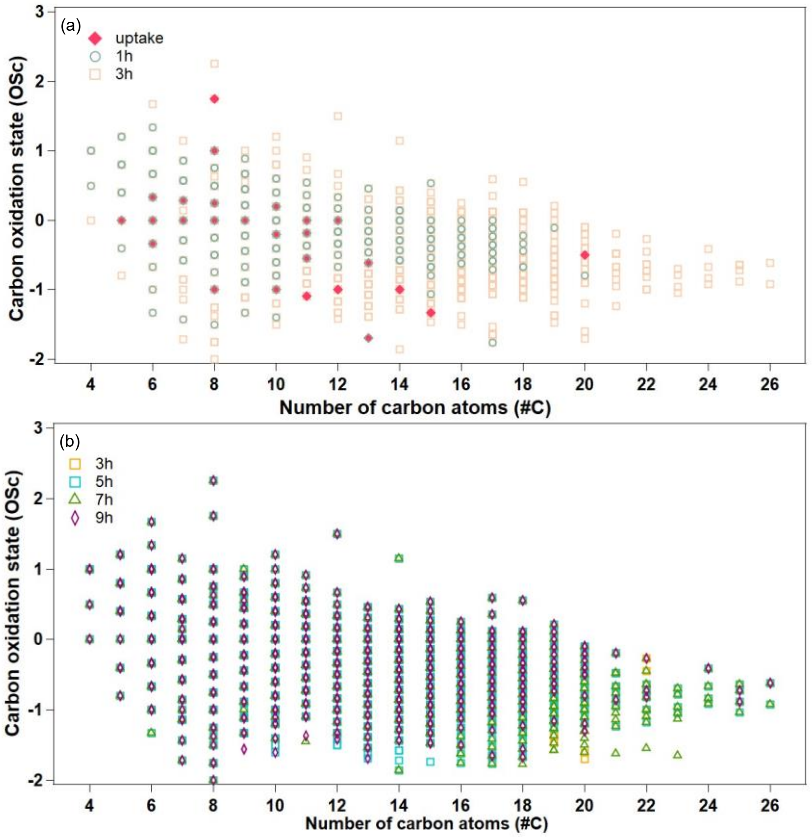

Supplementary Figure 10. Carbon oxidation state vs carbon number for detected CHO group ions (measured by Particle-EESI). Colored symbols denote different aqueous-phase reaction times. Data are split into (a) and (b) for better readability. Due to the long time of the experiment (9 hours), excess oxidation may leads to fragmentation.

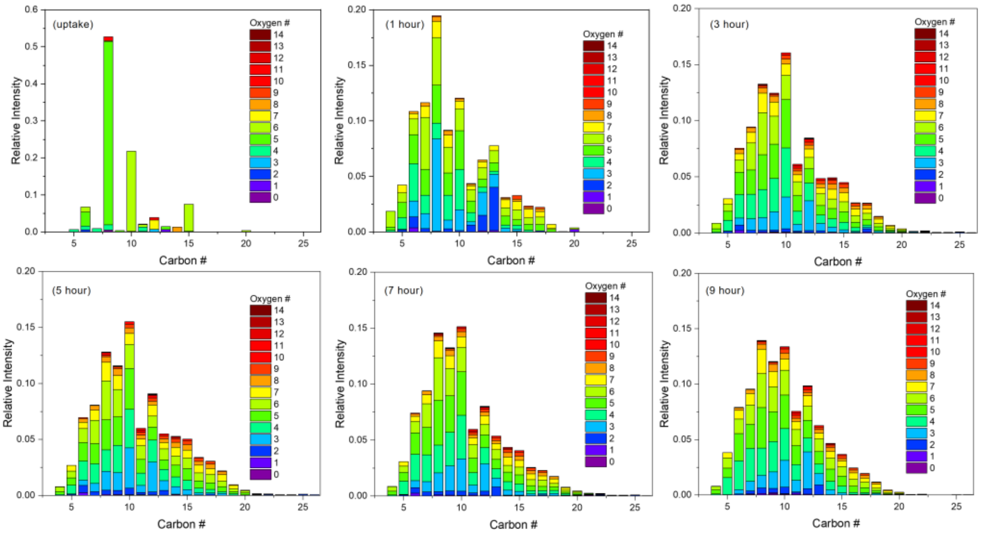

Supplementary Figure 11. The average carbon and oxygen distributions of aqSOA for different times of cloud processing as measured by the Particle-EESI.

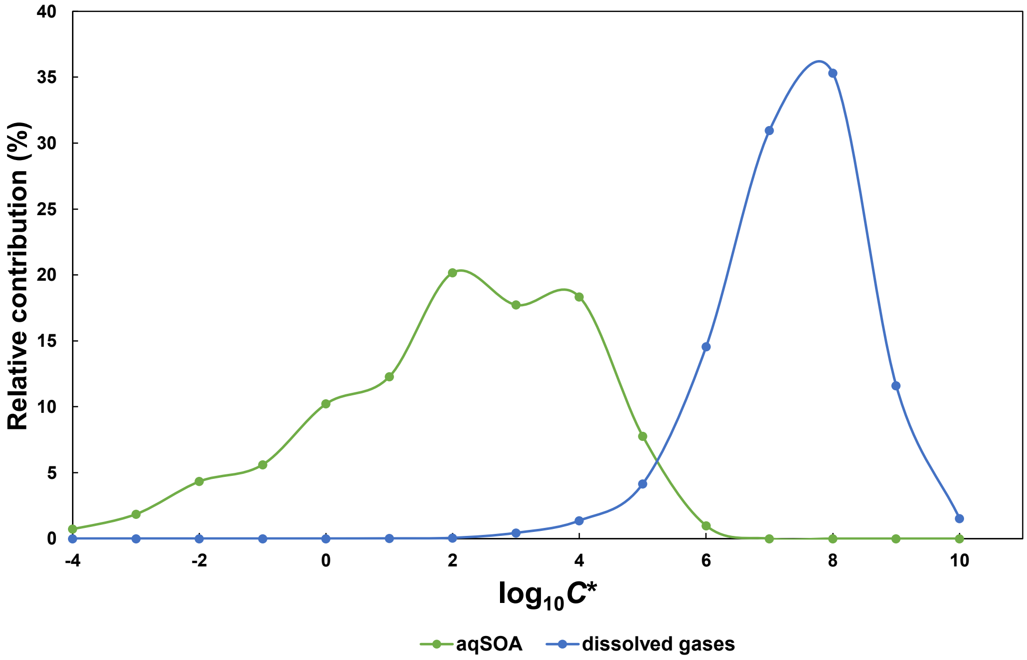

Supplementary Figure 12. Volatility distributions of the oxidation products of aqSOA phase (green line; measured by the Particle-EESI), and representative dissolved phase (blue line; calculated based on the gas concentrations measured by the Vocus and the Dual-EESI and calculated from the QEMRA model after 5 hours of experiment).

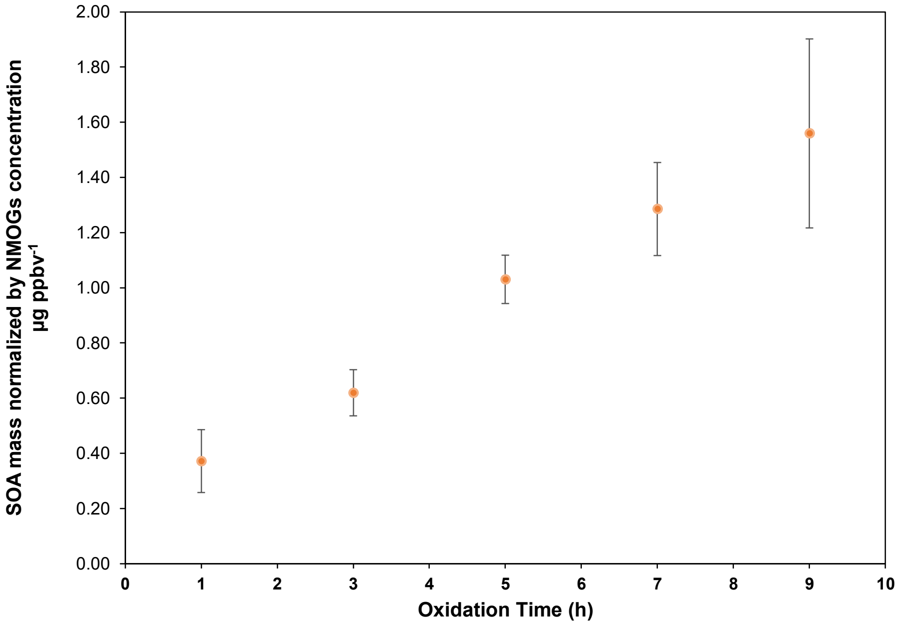

Supplementary Figure 13. Normalized average aqSOA mass as a function of the oxidation time. The error bars are the one standard deviation of the aqSOA mass calculated by two labeled isotopes.

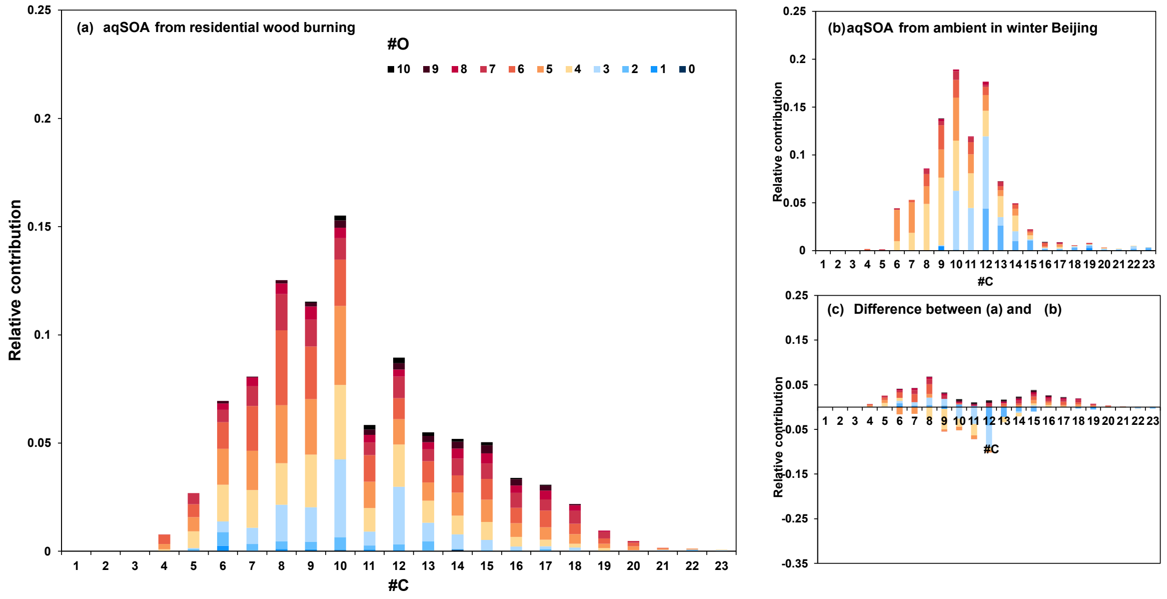

Supplementary Figure 14. Detailed chemical composition of the aqSOA. The average carbon number distribution (x-axis) colored by oxygen number. (a) AqSOA composition after 5 hours of cloud processing measured by the Particle-EESI after nebulization, (b) Beijing field campaign aqSOA composition measured by the same Particle-EESI. The differences between aqSOA and Beijing field campaign aqSOA are shown in c (positive signal means aqSOA is higher).

Supplementary Table 1. Summary of the WFR experiments for biomass burning emissions.

Exp. No.	Type	CO (ppmv)	CO_2 (ppmv)	THC (ppmv C)	MCE	RH (%)	T (K)	NO_x (ppmv)
Uptake-1	stove	69.4	770.2	30.13	0.92	98.2	293	670
Uptake-2	stove	49	838	16.4	0.94	98.8	294.6	N/A
1 hour oxidation	stove	112.6	1751.2	32	0.94	97.6	291.4	715
3 hour oxidation	stove	81.3	1134	29.5	0.93	98.1	293.1	685
5 hour oxidation	stove	78.9	837.7	31.5	0.91	98.2	291.7	465
7 hour oxidation	stove	75.4	1159.7	26.9	0.94	99.1	292.6	445
9 hour oxidation -1	stove	91.2	1110.9	24	0.92	100	292.6	490
9 hour oxidation -2	stove	41.2	745.8	16.1	0.95	99.1	293.3	N/A

Supplementary Table 2. Composition classes and the values for saturation mass concentration parameterizations.

Classes	n_C^0	b_C	b_O	b_{CO}	b_N
CH	23.8	0.4681			
CHO	22.66	0.4481	1.656	-0.7790	
CHN	24.59	0.4066			0.9619
CHON	24.13	0.3667	0.7732	-0.0779	1.114

Supplementary Table 3. List of organic ions detected, compound assignment, and their Henry law constants calculated by EPI.

m/z	Ion formula	Tentative compound assignment	$\log_{10}(H)$ based on EPI
31.0178	CH3O+	formaldehyde	1.03
33.0335	CH5O+	methanol	2.37
39.02	C3H3+	isoprene fragment	NAN
41.038578	C3H5+	propyne	-1.21
43.054226	C3H7+	propene	-2.18
45.033489	C2H5O+	acetaldehyde	1.17
47.012756	CH3O2+	formic acid	3.12
47.049141	C2H7O+	ethanol	2.25
53.038578	C4H5+	vinylacetylene	-1.29
55.054	C4H7+	1,3-Butadiene	-1.89
57.033489	C3H5O+	acrolein	1.45
57.069878	C4H9+	1-butene	-2.31
59.049141	C3H7O+	acetone	1.3
59.049141	C3H7O+	propanal	1.04
61.028404	C2H5O2+	acetic acid	3.26
63.044056	C2H7O2+	ethylene glycol	3.88
67.05423	C5H7+	cyclopentadiene	-1.8
69.034042	C4H5O	furan	-0.73

69.069878	C5H9+	isoprene	-2.09
69.069878	C5H9+	cyclopentene	-1.7
71.049141	C4H7O+	2-butenal	1.25
71.049141	C4H7O+	methyl vinyl ketone	1.58
71.085526	C5H11+	pentenes & methylbutenes	-2.5
73.028404	C3H5O2+	methylglyoxal & 2-oxopropanal	3.54
73.064789	C4H9O+	butanal	0.92
73.064789	C4H9O+	methyl ethyl ketone	1.18
77.023323	C2H5O3+	glycolic acid	4.07
79.05423	C6H7+	benzene	-0.73
81.069878	C6H9+	1,3-cyclohexadiene	-1.93
81.07	C6H9+	terpenes (α-pinene) fragment	NAN
83.049141	C5H7O+	2-methylfuran	-0.77
83.085526	C6H11+	cyclohexene	-1.82
85.028404	C4H5O2+	2-Butenedial	4.68
85.064789	C5H9O+	3-methyl-3-butene-2one	1.39
85.101173	C6H13+	(E)-hex-2-ene	-2.63
87.04406	C4H7O2+	butane-2,3-dione	3.7
87.080444	C5H11O+	butanone_2_3methyl	1.06
87.080444	C5H11O+	Pentanal	0.8
89.023323	C3H5O3+	pyruvic acid	5.66
89.059708	C4H9O2+	methyl propanoate	0.63
91.038971	C3H7O3+	lactic acid	3.95
91.075356	C4H11O2+	butanediols	3.64
93.054619	C3H9O3+	glycerol	5.2
93.069878	C7H9+	toluene	-0.77
95.049141	C6H7O+	phenol	3.25
95.085526	C7H11+	norbornene	-1.59
97.028404	C5H5O2+	furan-2-carbaldehyde	1.87
97.064789	C6H9O+	2,5-dimethylfuran	-0.82
97.101173	C7H13+	1-methylcyclohexene	-2.02
99.04406	C5H7O2+	furfuryl alcohol	3.66
99.080444	C6H11O+	hexanals	1.35
99.080444	C6H11O+	dimethyldihydrofuran (cyclohexanone)	1.29
101.09609	C6H13O+	hexanals	0.93
103.07536	C5H11O2+	pentanoic acid	2.89
105.05462	C4H9O3+	hydroxybutyric acid	3.82
105.06988	C8H9+	styrene	-0.44
105.091	C5H13O2+	methyl tertiary butyl ether	-0.3
107.04914	C7H7O+	benzaldehyde	1.87
107.08553	C8H11+	ethylbenzene	-0.9
107.08553	C8H11+	o -Xylene	-2.81
109.06479	C7H9O+	cresol (methylphenol)	3.21
111.04406	C6H7O2+	methylfurfural	1.83
113.05971	C6H9O2+	cyclohexanedione	3.82
113.09609	C7H13O+	ethylcyclopentanone	1.17

113.13248	C8H17+	octanal	0.43
115.11174	C7H15O+	heptanal	0.55
115.11174	C7H15O+	2,4 -dimethyl - 3 -pentanone	0.81
117.01823	C4H5O4+	fumaric acid	8.87
117.05462	C5H9O3+	5-Hydroxymethyl tetrahydro 2-furanone	5.3
119.04914	C8H7O+	benzofuran	0.28
119.07027	C5H11O3+	3-methyl-2,3,4- trihydroxy-1-butene	4.18
119.08553	C9H11+	methyl styrenes	-0.48
121.04954	C4H9O4+	2-methylglyceric acid	5.25
121.06479	C8H9O+	Tolualdehydes	1.83
121.10117	C9H13+	Mesitylen	-0.85
123.04406	C7H7O2+	2-hydroxybenzaldehyde	2.75
123.08044	C8H11O+	methyl anisol	0.45
125.02332	C6H5O3+	hydroxy benzoquinone	5.82
125.05971	C7H9O2+	guaiacol	4.78
125.05971	C7H9O2+	2-methoxyphenol	4.48
127.03897	C6H7O3+	5 -(hydroxymethyl) - 2 -furfural	6.26
129.06987	C10H9+	naphthalene	0.43
131.03389	C5H7O4+	dioxopentanoic acida	8.57
133.06479	C9H9O+	methylbenzofuran	0.24
133.10118	C10H13+	ethyl styrene	-0.76
133.10118	C10H13+	methylpropenylbenzenes	-0.83
135.04405	C8H7O2+	isophthalaldehyde	3.83
135.06519	C5H11O4+	deoxyribose	8.11
135.08044	C9H11O+	3 -methylacetophenone	2
137.05971	C8H9O2+	methyl benzoic acid	1.46
139.07536	C8H11O2+	4-(2-hydroxyethyl)phenol	7.52
141.05463	C7H9O3+	3-methoxycatechol	8.46
143.03389	C6H7O4+	1,2,3,4-benzenetetrol	8.02
143.08553	C11H11+	methylnaphthalene	0.24
145.04953	C6H9O4+	dimethylmaleic acid	8.48
147.04405	C9H7O2+	indene-1,3-dione	5.22
147.06519	C6H11O4+	methylglutaric acid	8.02
147.08044	C10H11O+	dimethylbenzofuran	0.19
149.02332	C8H5O3+	phthalic anhydride	2.2
149.04445	C5H9O5+	hydroxyglutaric acid	12.58
149.05971	C9H9O2+	cinnamic acid	4.89
149.09608	C10H13O+	methyl chavicol (=estragole)	0.34
151.07536	C9H11O2+	4-vinylguaiacol	4.77
153.05463	C8H9O3+	vanilin	7.08
153.06987	C12H9+	acenaphthylene	1.26
153.091	C9H13O2+	4-ethyl-2-methoxyphenol	4.31
153.1274	C10H17O+	camphor	1.15
155.07027	C8H11O3+	syringol	5.71
155.08553	C12H11+	1,1-biphenyl	0.38
155.14304	C10H19O+	cineole	0.69

157.10118	C12H13+	1,3-dimethylnaphthalene	0.19
157.15869	C10H21O+	decanal	0.18
159.06519	C7H11O4+	terebic acid	5.23
161.04445	C6H9O5+	ring fragment	10.54
161.08084	C7H13O4+	pimelic acid	7.89
165.05463	C9H9O3+	caffeic aldehyde	10.76
165.091	C10H13O2+	isoeuginol	4.57
165.091	C10H13O2+	2-methoxy-4-[(E)-prop-1-enyl]phenol	4.57
167.07027	C9H11O3+	1-(4-hydroxy-3-methoxyphenyl)ethanone	7.22
167.10666	C10H15O2+	2-methoxy-4-propylphenol	4.2
169.04953	C8H9O4+	vanillic acid	9.18
169.08592	C9H13O3+	2,6-dimethoxy-4-methylphenol	5.67
171.06519	C8H11O4+	a-oxy-syringol	8.69
171.10158	C9H15O3+	Ketolimononaldehyde (3-Acetyl-6-oxoheptanal)	7.14
177.07574	C7H13O5+	hydroxy-dimethylglutaric acid	12.33
179.07027	C10H11O3+	coniferaldehyde	8
181.08592	C10H13O3+	2-methoxy-4-prop-2-enylphenol	4.32
183.06519	C9H11O4+	3-Hydroxy-4-methoxybenzoic acid	9.18
183.10158	C10H15O3+	4-ethyl-2,6-dimethoxyphenol	5.54
191.05501	C7H11O6+	1,2,4-butanetricarboxylic acid	13.15
195.10158	C11H15O3+	allylsyringol	2.84
199.0601	C9H11O5+	syringic acid	10.4

Supplementary Table 4. Summary of the aqSOA yield calculations.

Exp. No.	aqSOA by AMS	SOA average mass	PTR VOC concentration	IVOC concentration	total NMOG concentration	total NMOG mass concentration	Gas Volume	SOA normalized mass	yield
**unit**	$\mu g\ m^{-3}$	µg	ppbv	ppbv	ppbv	$\mu g\ m^{-3}$	m^3	$\mu g\ ppbv^{-1}$	
**1.00**	7.0	113.2 ± 34.6	259.0	47.3	306.3	801.2	0.60	0.37 ± 0.11	0.43 ± 0.13
**3.00**	29.9	270.0 ± 36.6	370.6	67.6	438.3	1131.6	1.80	0.62 ± 0.08	0.23 ± 0.03
**5.00**	71.1	328.8 ± 27.9	271.4	49.5	320.9	836.1	3.00	1.03 ± 0.09	0.23 ± 0.02
**7.00**	173.3	369.7 ± 48.6	244.6	44.6	289.2	757.2	4.20	1.28 ± 0.17	0.21 ± 0.03
**9.00**	71.2	468.9 ± 103	255.8	46.7	302.5	806.2	5.40	1.55 ± 0.34	0.20 ± 0.04

Supplementary References

1. Graus M, Muller M, Hansel A. High resolution PTR-TOF: quantification and formula confirmation of VOC in real time. *J. Am. society Mass Spectrom* **21**, 1037-1044 (2010).

2. Jordan A, *et al.* A high resolution and high sensitivity proton-transfer-reaction time-of-flight mass spectrometer (PTR-TOF-MS). *Int. J. Mass spectrom.* **286**, 122-128 (2009).

3. Wang L, *et al.* Source characterization of volatile organic compounds measured by proton-transfer-reaction time-of-flight mass spectrometers in Delhi, India. *Atmos. Chem. Phys.* **20**, 9753-9770 (2020).

4. Wang L, *et al.* Characteristics of wintertime VOCs in urban Beijing: Composition and source apportionment. *Atmos. Environ.* **9**, 100100 (2021).

5. de Gouw J, Warneke C. Measurements of volatile organic compounds in the earth's atmosphere using proton-transfer-reaction mass spectrometry. *Mass Spectrom. Rev.* **26**, 223-257 (2007).

6. Cappellin L, *et al.* On quantitative determination of volatile organic compound concentrations using proton transfer reaction time-of-flight mass spectrometry. *Environ. Sci. Technol.* **46**, 2283-2290 (2012).

7. Krechmer J, *et al.* Evaluation of a New Reagent-Ion Source and Focusing Ion-Molecule Reactor for Use in Proton-Transfer-Reaction Mass Spectrometry. *Anal. Chem.* **90**, 12011-12018 (2018).

8. Lopez-Hilfiker FD, *et al.* An extractive electrospray ionization time-of-flight mass spectrometer (EESI-TOF) for online measurement of atmospheric aerosol particles. *Atmos. Meas. Tech.* **12**, 4867-4886 (2019).

9. Lee CP, *et al.* High-frequency gaseous and particulate chemical characterization using extractive electrospray ionization mass spectrometry (Dual-Phase-EESI-TOF). *Atmos. Meas. Tech.* **15**, 3747-3760 (2022).

10. Qi L, *et al.* A 1-year characterization of organic aerosol composition and sources using an extractive electrospray ionization time-of-flight mass spectrometer (EESI-TOF). *Atmos. Chem. Phys.* **20**, 7875-7893 (2020).

11. Stefenelli G, *et al.* Organic aerosol source apportionment in Zurich using an extractive electrospray ionization time-of-flight mass spectrometer (EESI-TOF-MS) – Part 1: Biogenic influences and day–night chemistry in summer. *Atmos. Chem. Phys.* **19**, 14825-14848 (2019).

12. Casotto R, *et al.* Chemical composition and sources of organic aerosol on the Adriatic coast in Croatia. *Atmos. Environ.* **13**, 100159 (2022).

13. Li Y, Pöschl U, Shiraiwa M. Molecular corridors and parameterizations of volatility in the chemical evolution of organic aerosols. *Atmos. Chem. Phys.* **16**, 3327-3344 (2016).

14. Donahue NM, Epstein SA, Pandis SN, Robinson AL. A two-dimensional volatility basis set: 1. organic-aerosol mixing thermodynamics. *Atmos. Chem. Phys.* **11**, 3303-3318 (2011).

15. Hodzic A, *et al.* Characterization of organic aerosol across the global remote troposphere: a comparison of ATom measurements and global chemistry models. *Atmos. Chem. Phys.* **20**, 4607-4635 (2020).

16. Lamkaddam H, *et al.* Large contribution to secondary organic aerosol from isoprene cloud chemistry. *Sci. Adv.* **7**, eabe2952 (2021).

17. Atkinson R, *et al.* Evaluated kinetic and photochemical data for atmospheric chemistry: Volume II–gas phase reactions of organic species. *Atmos. Chem. Phys.* **6**, 3625-4055 (2006).

18. Li K, *et al.* Uncovering the dominant contribution of intermediate volatility compounds in secondary organic aerosol formation from biomass-burning emissions. *National Science Review* **11**, nwae014 (2024).

19. Molteni U, *et al.* Formation of highly oxygenated organic molecules from aromatic compounds. *Atmos. Chem. Phys.* **18**, 1909-1921 (2018).

20. Whalley LK, *et al.* The influence of clouds on radical concentrations: observations and modelling studies of HO_x during the Hill Cap Cloud Thuringia (HCCT) campaign in 2010. *Atmos. Chem. Phys.* **15**, 3289-3301 (2015).

21. Griffith S, *et al.* OH and HO 2 radical chemistry during PROPHET 2008 and CABINEX 2009–Part 1: Measurements and model comparison. *Atmos. Chem. Phys.* **13**, 5403-5423 (2013).

22. Dusanter S, *et al.* Measurements of OH and HO 2 concentrations during the MCMA-2006 field campaign–Part 2: Model comparison and radical budget. *Atmos. Chem. Phys.* **9**, 6655-6675 (2009).

23. Herrmann H, *et al.* Tropospheric aqueous-phase chemistry: kinetics, mechanisms, and its coupling to a changing gas phase. *Chem. Rev.* **115**, 4259-4334 (2015).

24. Tong Y, *et al.* Quantification of solid fuel combustion and aqueous chemistry contributions to secondary organic aerosol during wintertime haze events in Beijing. *Atmos. Chem. Phys.* **21**, 9859-9886 (2021).
